# AI in phishing detection: a bibliometric review

**DOI:** 10.3389/frai.2025.1496580

**Published:** 2025-10-23

**Authors:** Daniela Popescul, Laura Diana Radu

**Affiliations:** Department of Accounting, Business Information Systems and Statistics, Faculty of Economics and Business Administration, “Alexandru Ioan Cuza” University, Iași, Romania

**Keywords:** phishing, social engineering, machine learning, artificial intelligence, deep learning, bibliometric review

## Abstract

**Background:**

Phishing represents a category of cyber-attacks based on social engineering, with a significant impact on individuals and organizations, and a high capacity for reinvention by adapting its modus operandi according to technological advancements. With a relatively simple scenario and without using sophisticated technologies, phishing attacks exploit user vulnerabilities, convincing them to disclose sensitive personal or organizational data. Within anti-phishing solutions, the detection of spoofed URLs, counterfeit websites, and email or other types of messages that lure the user into entering their data in a form, plays an important role. Against this backdrop, artificial intelligence (AI) technologies, particularly Machine Learning (ML), have been successfully employed in phishing detection, with a rich body of literature in this field.

**Objective:**

A review of the existing literature on phishing detection using AI was conducted. This study aims to fill this gap by providing comprehensive bibliometric analysis, complementing existing surveys in the field, focusing on the role of AI in phishing detection.

**Methods:**

A total of 1096 documents focusing on AI, ML, Deep Learning (DL), or Natural Language Processing (NLP) in phishing detection were extracted from the Web of Science (WoS) scientific database. The information from these documents was subsequently loaded into the Biblioshiny (Bibliometrix package) and VOSviewer software.

**Results:**

The dataset allowed for the identification of publication trends, influential documents and publications, patterns of author collaboration, and key topics of interest within the main author clusters. A thematic analysis of the field highlighted driving themes, niche themes, emerging and declining themes, and basic themes. Furthermore, thematic evolution over time was examined based on authors’ keywords. A thorough review of the most relevant articles identified through bibliometric analysis was conducted to discuss the primary methods of phishing detection using AI.

**Conclusion:**

The research field of AI in phishing detection has evolved significantly starting with 2016, with a focus on using ML algorithms to identify phishing websites by extracting discriminative features, and experienced a consistent growth in 2024. Recent work emphasizes a shift from classical ML to DL, the importance of feature selection and engineering, and the use of hybrid models and classifier stacking.

## 1 Introduction

In today’s digital age, the success of any organization—regardless of its size or sector—largely depends on how it understands and manages information. Information is not only a key operational resource, but also a strategic asset that influences decision-making, competitiveness, and organizational performance. When used effectively, information can improve the cost-efficiency of various internal processes and support the achievement of broader organizational goals.

Much like financial, human, or physical resources, information plays a central role in planning, coordination, and control. However, as organizations continue to generate and process increasing volumes of data, the management of this information becomes more complex. Without a clear understanding of the value and role of information, organizations risk exposing themselves to security threats, operational inefficiencies, and strategic blind spots.

An important step in ensuring the security of organizational information is the management of information assets. These assets—whether tangible or intangible—comprise collections of information that is worth protected (European Parliament and Council, 2022). Information assets are managed as distinct units, enabling them to be understood, shared, protected, and utilized effectively (The National Archives, 2017). Their value is tied not only to the advantages they bring to the organization, but also to the potential harm caused by loss, alteration, or unauthorized disclosure. Trust from stakeholders and business partners also depends on the integrity and availability of critical information (Popescul, 2014). Information, however, is not managed in isolation. The information assets are handled by people across the organization—employees, managers, and contractors—each with varying levels of access, awareness, and technical competence. This diversity introduces a significant variable: human behavior. Individuals may unintentionally become sources of risk through negligence, lack of training, or failure to follow established procedures. In some cases, poor judgment or susceptibility to manipulation can lead to serious breaches. The human factor, therefore, represents both a critical asset and a potential vulnerability.

The security of information assets involves minimizing vulnerabilities and addressing threats that could compromise confidentiality, integrity, or availability. In recent years, threat landscapes have become increasingly dynamic and diverse. Reports from the European Union Agency for Cybersecurity (ENISA) highlight top cybersecurity threats such as ransomware, malware, social engineering, data breaches, distributed denial-of-service (DDoS) attacks, information manipulation, and supply chain attacks. Although, in response to these evolving threats, data security has significantly improved through advanced technical solutions, the human element remains an essential and often vulnerable link in the information lifecycle, as knowledge and information are frequently handled, processed, or interpreted by people rather than machines. In effect, protecting organizational information is as much a human challenge as it is a technical one (Oprea, 2007). Social engineering exploits this human factor by manipulating individuals to disclose sensitive information, making it a particularly dangerous and evolving threat in the digital era.

In cybersecurity, the term **social engineering** refers to a wide range of activities that attempt to exploit user behavior in order to gain access to information or services that the attacker is not authorized to use. The users are lured “into opening documents, files or emails, visiting websites or granting unauthorized persons access to systems or services” (ENISA, 2023b). Within social engineering, **phishing** is a form of criminal activity in which the attacker obtains sensitive data, such as login credentials for banking applications or e-commerce platforms, credit card information, bank account details, Personal Identification Numbers (PINs), as well as other personal and/or confidential information, by using techniques to manipulate the identity of a person or organization (Xiang et al., 2011; Adebowale et al., 2019; Alsariera et al., 2020; Capuano et al., 2022). In Basit et al. (2021), phishing websites are considered frequent gateways for online social engineering attacks. Phishing is particularly dangerous because it has a direct impact on the physical world (Alsariera et al., 2020), with consequences such as drained bank accounts, compromised security systems, or even threats to personal safety. By deceiving individuals into sharing private information, phishing blurs the line between the digital and physical realms, making its effects far-reaching and tangible. In phishing, attackers often use psychological manipulation techniques. For example, an individual whose profile has been accurately determined through data collection from various social media platforms is sent an unusual request, outside the norms of internal procedures, as if coming from an official or superior with whom the targeted person does not usually have direct contact. The victims are led to believe there is an alleged urgency, they are flattered, promised rewards, and asked to maintain confidentiality, ultimately being guided into performing actions they would not normally do. Karim et al. also state that by using “social engineering tricks” the message can deceive the recipient into acting in the attacker’s favor, even without the need for malicious links or attachments sent digitally (Karim et al., 2019).

The trend of “escalating frequency, severity, and impact” associated with phishing, mentioned in 2019 by Adebowale et al. (2019) has continued in the following years, with the diversification of methods targeting victims and the increasing quality of attacks. According to ENISA, Europol and the FBI report that phishing and social engineering remain the main vectors for payment fraud, growing over time both in volume and sophistication (ENISA, 2023a). Beyond the costs incurred by organizations and individuals in managing it, phishing is used alongside identity theft (Gangavarapu et al., 2020) and ransomware. Currently, healthcare is one of the most targeted sectors by phishing (Sharma et al., 2023). In an specialized ENISA report on health sector, the scenario for an initial attack is described as starting with a phishing campaign, followed by a ransomware attack with negative effects on patient data (ENISA, 2023b).

The forms of “bait” for users have also evolved over time. Alongside technological advancements, email messages with numerous spelling mistakes were joined by SMS messages, social media posts, voice calls made by humans and later by synthetic voices, deep-fake video images, QR codes, and tampered mobile apps. Depending on how the user is deceived, there are various types of phishing. The “classic” *phishing* variant involves creating a website as a replica of a legitimate one and luring the victim to access it through email messages which contain a hyperlink with a URL similar to that of the original site. In the case of *pharming*, the victim is automatically redirected to the duplicate website, directly through DNS manipulation or execution of malicious code, making further “deception” unnecessary. *Smishing* refers to the type of phishing in which victims’ financial or personal information is collected with SMS messages. *Vishing* is a combination of phishing and voice, where information is provided over the phone by victims deceived through social engineering techniques. In *quishing*, the victim is directed to a malicious site or file by scanning a QR code. *Covert redirect* is a type of phishing attack that exploits vulnerabilities in third-party authentication systems, to redirect users to malicious websites without their knowledge. Unlike traditional phishing attacks, covert redirect does not require users to enter their credentials directly into a fake login page; instead, it tricks them into granting permissions to a malicious app or site, which then gains unauthorized access to their data or accounts. The attack is difficult to detect because it appears to be part of the legitimate authentication process. *Clone phishing* is a type of phishing attack where the attacker copies or “clones” a legitimate, previously sent email, typically one that contains a link or attachment. The attacker then alters the email by replacing the original link or attachment with a malicious version and sends it from an email address that appears to be from the original sender. Since the recipient is already familiar with the email content, they are more likely to trust and click the malicious link or open the attachment, leading to credential theft or malware installation.

The accuracy with which victims are targeted has also increased over time. *Spear-phishing* is a more sophisticated version of phishing that addresses specific organizations or individuals, about whom the attackers gather information in advance. This type of phishing often bypasses the detection power of automatic anti-phishing filters, as the approach, appearance, and content of the messages are much more personalized. Spear-phishing can be used to generate Advanced Persistent Threats (Karim et al., 2019). *Whaling* is a sub-type of spear-phishing, which addresses senior executives with high-level access by impersonating a trusted entity, such as the company’s CEO or a legitimate business partner. These attacks often present an urgent issue affecting the entire company or a critical customer complaint, pressuring the executive to act quickly (Karim et al., 2019). *Social phishing* and *context-aware phishing* are two techniques that use publicly available personal information to make the attacks more effective (De La Torre Parra et al., 2020).

## 2 Related works

As presented above, phishing attacks have evolved alongside technological advancements. Initially targeting computers, these attacks have progressively shifted toward mobile devices and IoT systems, leveraging social media, e-commerce platforms, and other online environments that attract large user populations. In Dwivedi et al. (2023), it is shown that criminals can exploit even the metaverse for phishing attacks by creating fake versions of real-world brands, tricking users into sharing personal information or sending cryptocurrency to counterfeit entities. In the ENISA report published in 2023, it is stated that current innovations in social engineering are primarily driven by AI, especially considering the release of ChatGPT during the reporting period. AI is used to create more convincing phishing emails and messages that closely mimic legitimate sources, while deepfakes are mainly employed for voice cloning. Deepfakes target the integrity and availability of data, introducing substantial risks to decisions based entirely on unverified data. For instance, a deepfake voice call led to a fraudulent bank transfer of nearly $35 million (ENISA, 2022). In Gupta et al. (2023), the authors describe how ChatGPT can be used to generate messages for spear-phishing. Its ability to learn communication patterns associated with, for example, a specific website or individual increases the likelihood that the generated messages will be credible and convincing, leading to the attacker obtaining the desired information in response.

In combating phishing, two major categories of solutions can be identified: educating users to increase awareness about the value of information as an intangible asset of their employer and of their own personal, financial, and medical data, and enhancing their understanding of how internet technologies work; and implementing technical solutions, such as: anti-phishing plug-ins or toolbars in browser, anti-malware software, visual similarity/content-based filtering, blacklist/whitelist-based methods, heuristics, Machine Learning (ML) and hybrid approaches (Sahingoz et al., 2019; Adebowale et al., 2019; Ali and Ahmed, 2019; Gangavarapu et al., 2020). Traditional technical solutions reflect a common trend in cybersecurity: attackers frequently exhibit greater innovation and technological expertise compared to their victims, as well as compared to law enforcement, researchers, and other professionals. For example, in Ali and Ahmed (2019), the authors present the inefficiency of blacklist-based methods, as these methods are outpaced by the speed—often measured in seconds—at which attackers create new websites. Also, list-based detection mechanisms involve frequent updates of URLs/IPs and significant system resources (Sahingoz et al., 2019). The great variety of phishing forms makes detection difficult.

As in many other fields, AI has begun to be utilized in cybersecurity, with the combination of human efforts and AI applications being considered by some authors as the only solution to address the escalation of attacks (Capuano et al., 2022). As Grover et al. (2023) highlight, the rise in manipulation attacks—enabled by advances in generating deceptive texts, images, audio, and even video (e.g., deepfakes)—requires equally advanced, automated detection methods. The increasing availability of datasets related to such attacks, along with improvements in computational capabilities, has accelerated the development of AI-based solutions for identifying and responding to phishing attempts more effectively and at scale. Security professionals are now considering AI not just as a technological trend, but as a necessary component in building resilient, adaptive defense systems in the face of a rapidly evolving threat landscape. In the greater AI sphere, ML is defined as the development of algorithms and statistical models that enable computers to perform tasks without explicit instructions, Deep Learning (DL) is a subset of ML that uses neural networks with many layers (known as deep neural networks) to model complex patterns in data, and Natural Language Processing (NLP) is the field in which machines are able to understand, interpret, and generate human language in a meaningful way (Santos et al., 2024). AI can identify spam, phishing, spear-phishing, and various other types of attacks by leveraging prior knowledge from datasets (Basit et al., 2021). Solutions based on AI have proven to be highly promising (Karim et al., 2019; Alsariera et al., 2020; Gangavarapu et al., 2020), but not infallible. Among their limitations, in Alsariera et al. (2020) are mentioned the “high false alarm rate, low detection rate, and the inability of single classifiers and some hybridized methods to produce highly effective and efficient phishing website detection solutions.” In contrast to opaque, black-box solutions, eXplainable AI (XAI) applications have been employed. Clarifying why an message is flagged as phishing is highly valuable, XAI in this area helps people recognize and avoid an ever-present threat (Capuano et al., 2022).

In response to these evolving threats and the increasing interest in AI-based security solutions, a growing body of research has emerged focusing on the use of ML and AI to detect phishing attacks. However, despite this surge in publications, there is a lack of comprehensive overviews that map the structure, development, and key contributions within this research domain. Our study therefore fills this gap by systematically analyzing the field through bibliometric techniques.

Previous bibliometric analyses and literature reviews on AI for phishing detection have yielded significant insights into the field. To contextualize the contribution of our research, we present the most relevant papers and their impact on advancing knowledge in this domain in Table 1.

**Table 1:** Previous findings in the field.

Study	Aim	Methods	Data	Main results
*Email classification research trends: review and open issues* (Mujtaba et al., 2017)	Review of e-mail classification methods from 2006 to 2016, analyzing five aspects: application areas, datasets, feature spaces, classification techniques, and performance measures	Comprehensive review and analysis	98 articles (56 articles from Web of Science core collection databases and 42 articles from Scopus database)	The authors identify five techniques—supervised, semi-supervised, unsupervised, content-based, and statistical learning—with supervised ML being the most common and Support Vector Machine showing the best performance, followed by Decision Trees and Naive Bayes
*A recent review of conventional vs. automated cybersecurity anti-phishing techniques* (Qabajeh et al., 2018)	Examination of the effectiveness of traditional anti-phishing approaches, such as awareness campaigns, user education, and periodic training sessions, in comparison to computerized anti-phishing techniques	Classification of anti-phishing approaches in the analyzed literature into 3 main categories: “education and legal, computerized using human-crafted methods, and intelligent ML methods”	75 studies	ML and rule induction are particularly effective in phishing prevention, offering high detection accuracy and easily interpretable results. The tendency to use ML and DL algorithms for website classification to identify phishing sites was considered promising by the authors for reasons of cost and accuracy
*Evaluation of phishing techniques based on machine learning* (Kunju et al., 2019)	Survey of phishing attacks and their detection methods, with the intention to raise user awareness about the associated risks, and present various machine learning techniques (kNN, Naïve Bayes, Decision Tree, SVM, Neural Network, Random Forest) used for predicting and preventing phishing websites	Overview of ML algorithms for detecting phishing websites, including k-Nearest Neighbors (kNN), Naïve Bayes, Decision Trees, Support Vector Machines (SVM), Neural Networks, and Random Forest	14 studies	The necessity of employing multiple techniques to enhance phishing detection effectiveness is highlighted
*Toward the detection of phishing attacks* (Athulya and Praveen, 2020)	The paper aims to raise user awareness about phishing strategies and present a hybrid detection method that offers fast response time and high accuracy	Review of various phishing attacks, evasion techniques, and anti-phishing approaches	9 research articles	The most effective approach to mitigating phishing attacks is raising user awareness and selecting the most appropriate anti-phishing security software
*Toward a systematic description of the field using bibliometric analysis: Malware evolution* (Mat et al., 2021)	A bibliometric analysis of a decade of evolution in malware research, considering it as an umbrella term for all malicious software, with a specific focus on Android malware due to a significant rise in occurrences in 2019	Bibliometric review	1,278 articles	The article does not explicitly address phishing
*Applications of deep learning for phishing detection: A systematic literature review* (Catal et al., 2022)	Analysis of the use of DL for phishing detection	Systematic literature review	43 studies	The most commonly used algorithm is the Deep Neural Network (DNN), followed by Convolutional Neural Networks (CNN) and Recurrent Neural Networks (RNN)/Long Short-Term Memory Networks (LSTM). The study also indicates that DNN and Hybrid DL algorithms achieved the best performance in phishing detection
*A bibliometric analysis of phishing in the Big Data era: high focus on algorithms and low focus on people* (Pejić-Bach et al., 2023)	A co-occurrence analysis using VOSviewer on a set of 136 articles focused on big data and phishing	Bibliometric review	NA (WoS database)	Predominantly technical research (computer science, engineering, telecommunications); big data ML cluster emphasizes ML/DL benefits for real-time anti-phishing; approaches include models, voting frameworks, consensus clustering, URL analysis; Gray Wolf Optimizer outperforms other algorithms via feature analysis (e.g., URL length, HTTP response)
*A systematic literature review on phishing website detection techniques* (Safi and Singh, 2023)	An update in the previous systematic literature surveys with more focus on the latest trends in phishing detection techniques	Systematic literature review	80 scientific papers published between 2017 and 2021	ML techniques dominated phishing detection (71.25%), followed by heuristic (66.25%), visual similarity (43.75%), DL-based (17.5%), and list-based methods (12.5%); PhishTank was the main data source, while Random Forest, SVM, and Decision Tree were the most used ML algorithms, with CNN achieving the highest accuracy (99.98%)
*Mapping the phishing attacks research landscape: a bibliometric analysis and taxonomy* (Mutluturk and Metin, 2023)	A holistic approach of the topic and presentation of an in-depth analysis of phishing research from 2004 to 2023, emphasizing the field’s steady growth, emerging trends, and collaborative networks	Bibliometric review	3,139 phishing-related articles indexed in the Web of Science database	ML-based techniques play a central role in phishing research, with CANTINA+ (Xiang et al., 2011) ranking 3rd among the Top 10 Most Cited Publications (2004–2013), after studies by Jagatic et al. (2007) and one on the economics of information security (*Science*). In 2014–2023, Sahingoz et al. (2019) ranks 2nd, followed by Chiew et al. (2019) and Mahdavifar and Ghorbani (2019), highlighting the growing impact of AI-related approaches (Mutluturk and Metin, 2023). Zhang and Xiang emerge as key co-citation nodes, while Chiew leads another cluster; Cranor and Hong are the most cited authors. Among keywords, *Machine Learning* ranks 3rd and *Neural Networks* 12th
*Enhancing spear phishing defense with AI: a comprehensive review and future directions* (Mohamed et al., 2024)	A critical analysis of AI techniques, including ML, NLP, but also behavioral analytics, mitigating spear phishing attacks	Comprehensive review	30 seminal papers	ML models are effective for pattern recognition but require extensive training data, whereas NLP techniques enhance contextual and semantic understanding, improving detection of sophisticated phishing attempts

The usefulness of applying ML and DL techniques in phishing detection was recognized as early as 2017–2018, when several review studies were published to analyze their role in preventing email (Mujtaba et al., 2017) and website phishing (Qabajeh et al., 2018). In the following years, available algorithms were compared by various authors (Kunju et al., 2019; Athulya and Praveen, 2020), basing their analyses on a relatively small number of articles. Previous bibliometric studies have approached broader areas, such as malware in general (Mat et al., 2021), phishing in general (Mutluturk and Metin, 2023), and the relationship between phishing and Big Data (Pejić-Bach et al., 2023), without focusing on the use of AI in phishing detection.

Against this background, the present work has the following main objectives:

- To analyze publication trends, influential documents, and leading sources within the field of AI-driven phishing detection;
- To identify collaboration patterns and author clusters, thereby uncovering the structure of the research community;
- To perform a thematic mapping and evolution analysis using authors’ keywords to detect driving, emerging, declining, niche, and foundational themes over time;
- To provide a critical discussion of the most relevant articles, offering insights into the primary AI-based techniques employed for phishing detection;
- To identify and propose integration pathways for AI-powered phishing detection into organizations: Security Information and Event Management platforms, endpoint protection, secure email gateways, and cloud-based defense systems.

By addressing these objectives, the study aims to offer researchers, practitioners, and policymakers a clearer understanding of the field’s intellectual landscape, key developments, and future directions.

The remainder of this paper is organized as follows. Section 2 outlines the research methodology, including data sources, tools, and bibliometric techniques. Section 3 presents the results of the analysis. It covers publication trends, key documents and sources (3.1), collaboration networks and co-citation patterns (3.2), and the thematic development of the field (3.3). Section 4 discusses the main findings, with a focus on the AI techniques used in phishing detection. Section 5 concludes the paper by summarizing key contributions and suggesting directions for future research.

## 3 Research methodology

To conduct the study, we considered bibliometric analysis to be the most appropriate method. According to Donthu et al. (2021), this approach condenses a vast amount of bibliometric data to illustrate the current intellectual landscape and highlight emerging trends within a specific topic or field. It is particularly suitable when the scope of review is broad, and the data set is too extensive for manual review. In recent years, a significant number of researchers have utilized knowledge-mapping tools to examine developmental trends and the evolution of various disciplines and research fields. Due to the availability of advanced computational tools, this process has become far more accessible. These tools enable the statistical and quantitative analysis of a large number of publications and academic articles, facilitating the generation of descriptive statistics, the creation of keyword networks, and the establishment of connections between articles, publications, citations, authors, institutions, and countries (Gómez-Caicedo et al., 2022).

The aim of this paper is to identify, evaluate, and synthesize relevant studies on the use of AI in mitigating phishing. To understand the landscape of the field, the paper seeks to answer the following research questions:

*RQ1*. How has the publication landscape in AI for phishing detection research evolved over time?

*RQ2*. What are the core thematic clusters within the AI-based phishing detection research field, and how do these clusters interact and evolve over time?

*RQ3*. How are the AI technologies identified in the study utilized within organizations?

The research approach is structured and follows the PRISMA Reporting Guidelines for systematic reviews (Moher et al., 2009) aiming to ensure a rigorous evaluation of the literature published in the field. A comprehensive literature search was conducted using the Web of Science (WoS) database. The choice of this database is motivated by its multidisciplinary nature and reputation.

The search phrase included the following terms “artificial intelligence,” “AI,” “natural language processing,” machine learning,” “deep learning,” “phishing,” and “detection.” The search was restricted to articles written in English, with no limitation on the time frame. The document types included in the analysis were journal articles, conference proceedings, and book chapters. Using the *Refine results* option on the platform, we excluded documents from the following categories: early access papers, review articles, retracted publications, and data papers. The review articles were excluded to avoid duplicating information, as they summarize the findings of original articles, and their inclusion could lead to distortions in bibliometric analysis. Similarly, we excluded early access articles, as they have not yet been formally assigned to a specific volume or issue and may be subject to modifications before their final publication, and their consideration could introduce inconsistencies in the bibliometric analysis, as metadata such as the number of pages, citations, and affiliations may change. Furthermore, early access papers may occasionally appear as duplicates in databases, being listed both as early access and as the final published article. Since we have removed duplicates, this issue does not affect the results of the research; however, we consider it important to be mentioned. The search criteria are detailed in Table 2.

**Table 2:** Search criteria for extracting scientific articles from scientific database.

Keywords	((ALL = ((“artificial intelligence” OR “AI” OR “natural language processing” OR “machine learning” OR “deep learning”) AND phishing AND detection)))
Database	Web of Science
Exclusion criteria	Early Access or Data Paper or Retracted Publication or Review Article or Editorial Material (Exclude – Document Types) and Turkish and Spanish (Exclude – Languages)
Period	Unrestricted
Language	English
Search date	27 August 2025

**Figure 1:**
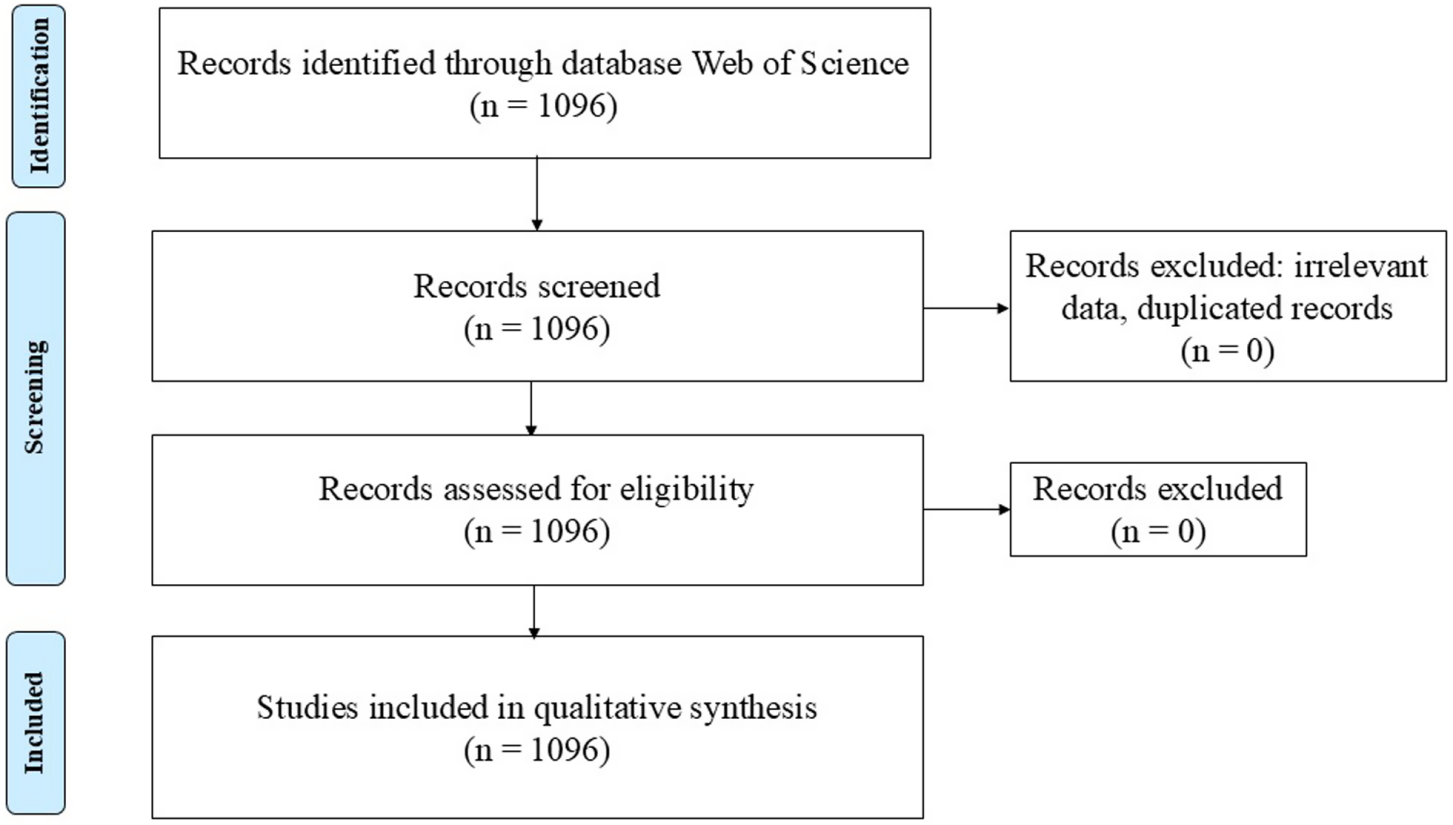
PRISMA 2009 flow diagram (Source: adapted from Moher et al., 2009).

The search yielded 1,096 documents. The relationship between research area, author(s), citation and impact journal was analyzed. Due to the export limitations of WoS, the operation was conducted in three stages: first for articles 1–500, next for articles 501–1,000 and subsequently for articles 1,001–1,096. The record content included full records and cited references. The selected export format was plain text. We manually addressed inconsistencies related to incomplete or missing data. We standardized the names of authors, journals, conferences, and publishers in cases of inconsistencies. No duplicates were identified. All articles were then merged into a single file and imported into the Biblioshiny (Bibliometrix package) and VOSviewer software packages. Figure 1 summarizes the study review protocol.

Biblioshiny and VOSviewer are two widely used tools for the bibliometric analysis of scientific output, each with its own strengths: Biblioshiny is more effective in visualizing trends, generating clearer and more easily interpretable graphics, while VOSviewer offers greater precision in clustering algorithms (Jia et al., 2022). They enable the creation of various networks, such as co-authorship, co-citation, and keyword co-occurrence, as well as the identification of the most influential publications and research. Using this information, it is possible to analyse the evolution of themes related to the use of AI in phishing detection, explore the connections between discussed topics, and identify emerging trends and areas for further development in the field.

The Bibliometrix package offers the necessary options for quantitative analysis of articles within a dataset, proving to be particularly useful when dealing with large volumes of data, where a comprehensive analysis would be impossible or, at the very least, highly challenging to perform. The package enables a wide range of analyses, such as co-citation analysis, examination of collaborations among authors, institutions, and countries, and the exploration of relationships between various keywords declared by authors or identified by algorithms implemented in bibliographic databases, among others.

The VOSviewer software includes advanced techniques for network layout and clustering and provides functionalities for analysing author collaborations, co-occurrence, citation, co-citations, bibliographic coupling, and, notably, the concepts used together. The application employs NLP to create term co-occurrence networks, automatically distinguishing between relevant and irrelevant concepts.

In this research, Biblioshiny (Bibliometrix package) was utilized to analyse the main data, identify the top ten most influential authors and journals, and determine the primary research directions and their evolution, while VOSviewer was used to examine author collaborations and co-citations. The descriptive insights generated through Biblioshiny were integrated with the advanced visualizations of VOSviewer to achieve a comprehensive and synergistic approach to the bibliometric review.

Table 3 presents the main information regarding the documents from the dataset generated by Biblioshiny based on our dataset extracted from WoS database. It includes 621 articles, 4 book chapters, and 471 conference proceedings, extracted from 644 sources and published between 2005 and 2025 by 3,327 authors. Out of the 1,096 documents, only 38 are single authored (~3.46%), with the remainder being collaborative works. The average number of authors per document is 3.84. The total number of citations received by the documents in the dataset is 13,435, with an average of 12.26 citations per published document. Additionally, 57% of the citations were concentrated on 77 articles, representing approximately 10% of the total documents analyzed. This indicates that a small proportion of articles have had a significant impact on the field. The total number of references in the dataset is 25,231. The annual growth rate during the examined period was 27.51%, with the growth particularly concentrated in recent years.

**Table 3:** The information about main data.

Description	Results
Main information about data	
Timespan	2005:2025
Sources (journals, books, etc.)	644
Documents	1,096
Annual growth rate %	27.51
Document average age	3.45
Average citations per doc	12.26
References	25,231
Document contents	
Keywords plus (ID)	296
Author’s keywords (DE)	2,378
Authors	
Authors	3,327
Authors of single-authored docs	37
Authors collaboration	
Single-authored docs	38
Co-authors per doc	3.84
International co-authorships %	28.1
Document types	2005:2025
Article	621
Book chapter	4
Proceedings paper	471

To achieve the aim of this study, we analyzed the most influential articles and publications in the field of AI-based phishing detection, as well as the key research directions, by identifying thematic areas and their evolution over time.

## 4 Results

The bibliographic analysis is divided into two components, following recommendations from the literature (Donthu et al., 2021): performance analysis and science mapping. Performance analysis involves examining the contributions of dataset constituents, such as authors, journals, institutions, and countries, and is descriptive in nature. It measures productivity through the number of articles published within a specific time frame, the impact through the number of citations, and the influence of research components by tracking citations per year, per article, and per journal (Chiroma et al., 2024). Science mapping focuses on analysing the relationships among the elements in the dataset, such as citation analysis, co-citation analysis, bibliographic coupling, co-word analysis, and co-authorship analysis (Donthu et al., 2021).

### 4.1 Publication trends, impactful documents and publications

**Figure 2:**
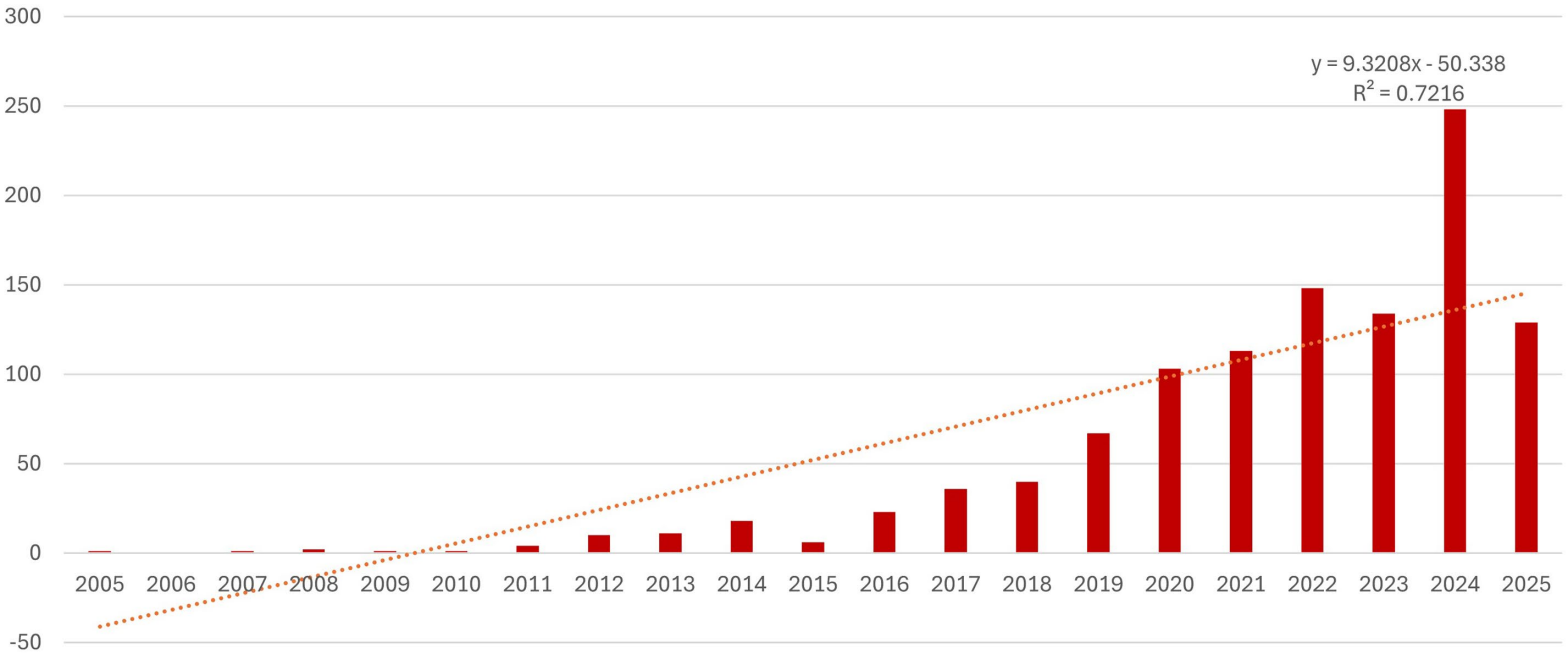
Annual quantitative distribution of publications.

Research on the use of AI in phishing detection has grown significantly in recent years, as expected, driven by the increase in computational power and innovations across all branches of AI. This trend confirms that phishing remains a persistent, adaptive, and challenging global threat, and the authors are in search of relevant solutions against it. In the first 10 years included in the analysis, the number of published articles on this topic was relatively small (49 documents). However, starting in 2016, the number of published documents began to increase at an accelerated pace, reaching a peak in 2024 when the number of published articles nearly doubled compared to the previous year (Figure 2).

Naturally, given that significant advancements in the field have occurred in recent years, the documents with the greatest impact, as measured by citation count, have generally been published in the past few years. Among the top 10 most-cited studies, the majority (9 out of 10) were published within the last 8 years (Table 4). This analysis was undertaken using Bibliometrix package.

**Table 4:** Top ten most impactful articles (papers sorted by local citations rate).

Title	Research area	LC	GC	LCR (%)
Toward detection of phishing websites on client-side using machine learning based approach (Jain and Gupta, 2018b)	ML	67	96	69.79
Detection of phishing websites using an efficient feature-based machine learning framework (Rao and Pais, 2019)	ML	78	133	58.65
Phishing website detection based on multidimensional features driven by deep learning (Yang et al., 2019)	DL	84	146	57.53
Machine learning based phishing detection from URLs (Sahingoz et al., 2019)	ML	180	323	55.73
PhishStorm: detecting phishing with streaming analytics (Marchal et al., 2014)	ML	70	132	53.03
A machine learning based approach for phishing detection using hyperlinks information (Jain and Gupta, 2019)	ML	57	114	50.00
A new hybrid ensemble feature selection framework for machine learning-based phishing detection system (Chiew et al., 2019)	ML	89	194	45.88
A stacking model using URL and HTML features for phishing webpage detection (Li et al., 2019)	ML	61	134	45.52
CANTINA+: a feature-rich machine learning framework for detecting phishing web sites (Xiang et al., 2011)	ML	135	324	41.67
A comprehensive survey of AI-enabled phishing attacks detection techniques (Basit et al., 2021)	–	61	163	37.42

LC, local citations; GC, global citations; LCR, local citations/global citations Ratio (%).

Local citations count the number of citations a document receives from other articles within the dataset, reflecting its influence within the analyzed field. Global citations count the number of citations received by a work across the entire WoS database, indicating its broader impact across various disciplines. The ratio between local citations and global citations reflects the level of specialization of each article in the dataset (Batista-Canino et al., 2023). A higher ratio of local citations suggests that the article is more specialized and highly relevant to the specific research area under investigation.

ML is the most frequently proposed approach for automated phishing detection, as evidenced by the top 10 most-cited articles in the field, presented in Table 4. In the analyzed dataset, among the articles in the top 10 by citation count, the article titled “Toward Detection of Phishing Websites on Client-Side Using Machine Learning-Based Approach” (Jain and Gupta, 2018b) ranks first with a 69.79% local citation ratio, indicating a very high level of specialization in the field. The second position is occupied by “Detection of Phishing Websites Using an Efficient Feature-Based Machine Learning Framework” (Rao and Pais, 2019) with a 58.65% ratio. Both papers propose phishing detection models based on ML algorithms. The first paper utilizes features extracted from URL functions, hyperlinks, CSS, authentication forms, and identity, while the second paper uses features extracted from URLs, website content, and third-party services. On third place is the paper entitled “Phishing Website Detection Based on Multidimensional Features Driven by Deep Learning” (Yang et al., 2019) with a 57.53% local citation ratio. The authors propose using DL for phishing detection. Their approach is multidimensional and involves two stages: in the first stage, features from the character sequence of the URL are extracted and used for rapid classification through DL; in the second stage, statistical features of the URL, page code features, web page text features, and results from the rapid DL classification are combined into a multidimensional features’ set, in order to increase detection accuracy.

Regarding the productivity and popularity of publications, approximately half of the articles (584) were disseminated through 132 journals, volumes, and books, whereas the remainder were published across an additional 512 distinct publications. This analysis was undertaken using Bibliometrix package. Table 5 presents the top 10 publications ranked by the number of citations. It also includes relevant information about these journals: H-index, G-index, M-index, the number of published documents (with their ranking based on the number of articles), the impact factor for 2024, JRC category, and quartiles based on WoS classification. Among the top ten journals, three are classified as Quartile 1 (Q1) and five as Quartile 2 (Q2). Quartiles (Q1 to Q4) represent the ranking tiers of journals within a given subdiscipline, with Q1 indicating the highest ranking. Since the topic is closely related to the ICT field, all journals are primarily categorized under computer science. *IEEE Access* journal published the most articles (78), and the articles in this journal had the highest number of citations (1,354). In second place for citations is *Computers & Security* with 662 citations, followed by *Expert Systems with Applications* with 627 citations. In terms of productivity, Computers & Security ranks second with 27 papers, followed by *Electronics* with 24 papers, although it is ranked 5th in terms of citations (168). *ACM Transactions on Information and System Security* stands out with a significant impact, having published just one article but receiving 324 citations. Other journals with a smaller number of articles but significant impact include *Telecommunication Systems* (4 articles and 294 citations), *Information Sciences* (4 articles and 262 citations), *Journal of Network and Computer Applications* (7 articles and 282 citations), and *Journal of Ambient Intelligence and Humanized Computing* (4 articles and 192 citations). In terms of research areas, the majority of publications (641) are from the field of Computer Science, followed by Engineering (267) and Telecommunications (179).

**Table 5:** Top ten journals publishing ranked by total citations.

Publications	h-index	g-index	m-index	TC	TC (%)	NP	RA	IF	Q
IEEE Access	21	35	2.33	1,354	10.08	78 (1)	CS, E, T	3.6	Q2
Computers and Security	14	25	1.08	662	4.93	27 (2)	CS	5.4	Q1
Expert Systems with Applications	8	13	0.57	627	4.67	13 (5)	CS, E, ORMS	7.5	Q1
Neural Computing and Applications	7	9	1.00	343	2.55	8 (9)	CS	4.5	Q2
Electronics	10	17	1.67	325	2.42	24 (3)	CS, E, P	2.6	Q2
ACM Transactions on Information and System Security	1	1	0.067	324	2.41	1 (120)	CS	2.6	Q2
Telecommunication Systems	3	4	0.375	294	2.19	2 (26)	T	2.3	Q3
Journal of Network and Computer Applications	3	7	0.200	282	2.10	7 (14)	CS	8	Q1
Information Sciences	4	4	0.333	262	1.95	4 (26)	CS	6.8	Q1
Journal of Ambient Intelligence and Humanized Computing	4	4	0.500	192	1.43	4 (26)	CS, T	3.6	Q2
Totals	–	–	–	5,349	–	170	–	–	–

CS, Computer Science; E, Engineering; ORMS, Operations Research and Management Science; T, Telecommunications; RA, Research area; Q, Quartile.

### 4.2 Collaboration network of author and co-citation

In this study, various bibliometric techniques were employed to analyse the research landscape related to using AI in phishing detection. The selection of indicators was guided by their relevance in capturing the impact, collaboration patterns, and thematic structure of the field. The scope and complexity of the analyzed field significantly limit the possibility of singular research efforts. Numerous research groups have proposed anti-phishing solutions developed using AI. Collaboration networks provide insights into the structure of research communities, key contributors, and interdisciplinary trends. Analysing co-authorship dynamics helps identify influential research groups and partnerships. To achieve this, we utilized VOSviewer software with the following criteria: a minimum of 3 documents per author, at least 5 citations per document to filter out weakly connected nodes and full counting method. A threshold of 3 documents per author was selected to focus on researchers with a relevant level of influence and to minimize the risk of including collaborations with a marginal impact. The minimum number of citations was set at 5 per document to consider only works with a reasonable influence in the field. Thresholds that are too low or too high can negatively affect the results, either by including occasional collaborations or by excluding relevant contributions. We considered these thresholds reasonable in relation to the topic and the size of the dataset. For example, other authors have set the minimum threshold at 5 documents per author and 10 citations per document for a dataset consisting of 4,875 papers (Ezugwu et al., 2021). To determine the thresholds, we conducted empirical tests on the dataset to achieve a set of stable and interpretable clusters. Thresholds that are too low or too high can negatively impact the results, either by including authors with insignificant impact or by excluding relevant contributions. To determine appropriate thresholds, we conducted empirical tests on the dataset to obtain a set of stable and interpretable clusters.

**Figure 3:**
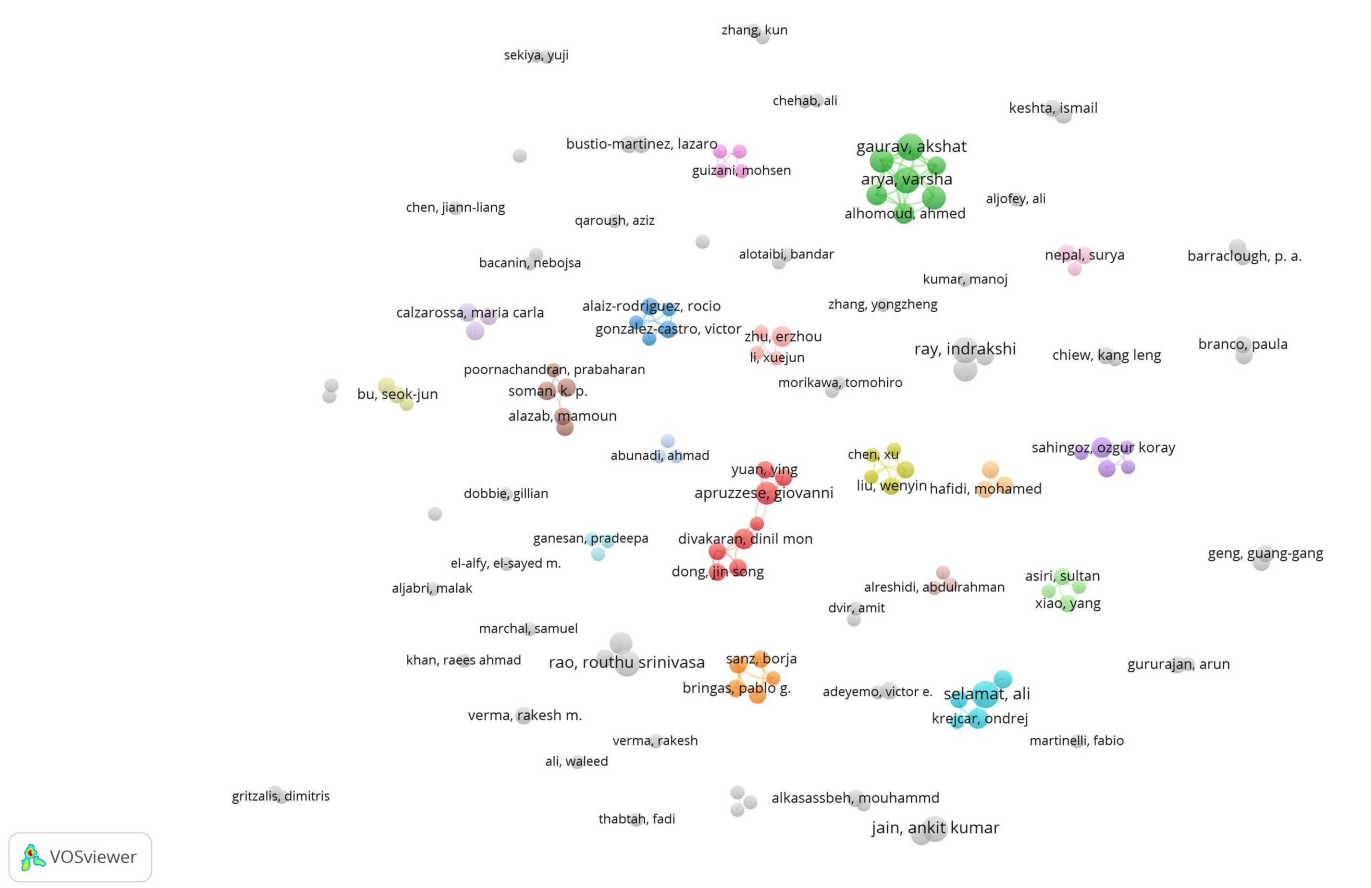
Co-authorship network for authors based on number of research.

In the VOSviewer network, each node represents an author, and the connections between nodes reflect the intensity of collaboration, determined by the number of articles they have co-authored. Four clusters were identified, consisting of five authors each, along with three clusters of four authors, three clusters of three authors, and ten clusters of two authors (Figure 3).

In terms of productivity, Akshat Gaurav (10) and Ali Selamat (10) have published the most papers in the dataset, followed by Varsha Arya (9), Ankit Kumar Jain (9), Routhu Srinivasa Rao (9) and Indrakshi Ray (9). Akshat Gaurav and Varsha Arya are in the same cluster. Regarding the topics covered, Gaurav research the integration of semantic web and AI technologies (DL recurrent neural network, CNNs) for robust phishing detection (Gaurav et al., 2024; Gupta et al., 2024b). Ali Selamat focusses on using ML techniques in phishing detection analysing the performance of various algorithms based on DL and NLP (Nguyet et al., 2021; Quang et al., 2021). Jain and Gupta have developed ML algorithms for phishing detection, with two of their papers appearing in the top 10 most-cited documents. The group with the most substantial connections consists of Akshat Gaurav, Varsha Arya, Kwok Tai Chui, Ahmed Alhomoud, Razaz Waheeb Attar, Shavi Bansal and Brij Bhooshan Gupta. They have published articles related to using ML for phishing detection trying to identify the most efficient model (Gaurav et al., 2024; Gupta et al., 2024a; Quang et al., 2021) and proposed optimized DL models for attack detection using feature selection techniques and hyperparameter optimization algorithms (Brown-Bear Optimization or Cuckoo Search), achieving high performance in detecting malicious URLs and attacks in web ecosystems. Another group, consisting of Igor Santos, Borga Sanz, and Xabier Ugarte-Pedrero, have published articles related to spam filtering methods based on anomaly detection using ML algorithms (Laorden et al., 2014; Santos et al., 2012). This topic is significant since spam messages are a common method for spreading computer viruses, worms, and phishing attempts, with statistics indicating that 46.8% of email traffic consists of spam messages (Petrosyan, 2024). Another research group, comprising Kutti Padanyl Soman, Ravi Vinayakumar, Prabaharan Poornachandran, Mamoun Alazab, and Xiaosong Zhang, has explored the advantages of DL in phishing detection and developed a framework for cyber threat situational awareness based on email and URL data analysis (Vinayakumar et al., 2019; Vinayakumar et al., 2019). Sultan Asiri, Yang Xiao, Saleh Alzahrani and Tieshan Li have also investigated the use of DL for phishing detection and created an anti-phishing system capable of identifying both regular phishing attacks and more specific threats such as Tiny Uniform Resource Locators (TinyURLs) and Browsers in the Browser (BiTB; Asiri et al., 2023; Asiri et al., 2024; Asiri et al., 2024).

**Figure 4:**
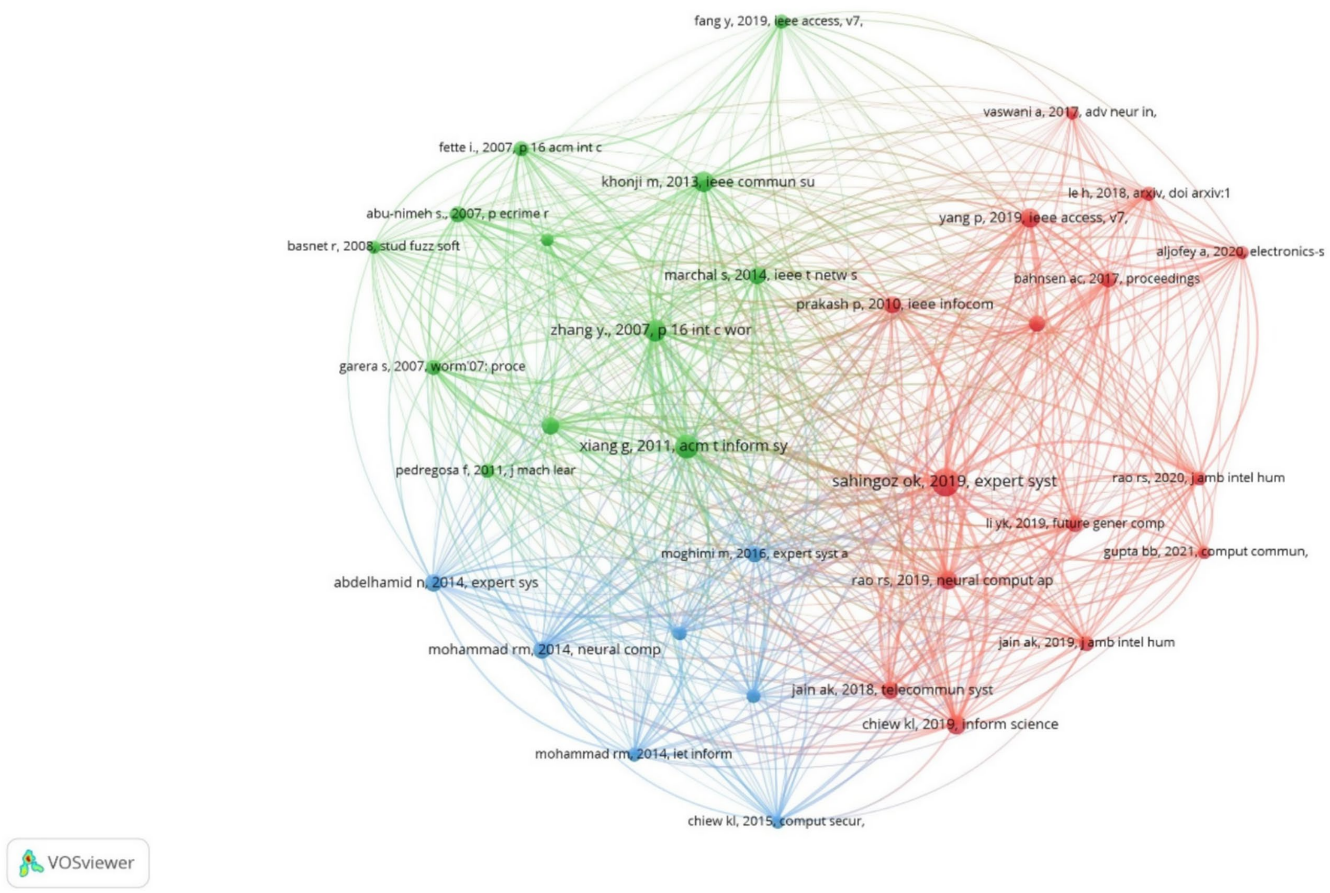
Co-citations network for references.

Another important indicator for the analyzed field is co-citation analysis. Citation metrics serve as a proxy for research impact, highlighting influential papers, authors, and journals. They help identify seminal works that have shaped the field over time. According to Donthu et al. (2021), publications that are frequently co-cited often exhibit semantic similarities, and co-citation analysis can lead to a better understanding of the fundamental themes within the field. Figure 4 presents a co-citation map for references with at least 40 citations to focus on impactful works and full counting method, generated by VOSviewer. A threshold of 40 citations per document was selected to highlight the articles with the greatest impact on the analyzed field. Our aim was to represent only the articles that have a defining influence on the topic researched. Similarly to the collaboration analysis, we conducted empirical experiments to identify the optimal and relevant minimum threshold. Co-citation network was mapped to visualize knowledge flows and intellectual foundations within the domain. Publications are connected when they appear in the reference list of another publication, with each connection representing a co-citation. The result includes 34 references grouped into 3 clusters and 1076 connections. Node size indicates the number of citations, the connection between two nodes shows that the references appeared together, and the thickness of the lines serves as an indicator of the frequency of these co-citations.

In the first cluster, the paper “Machine Learning Based Phishing Detection from URLs” (Sahingoz et al., 2019) ranks first with 180 citations and 33 links, followed by “Phishing Detection Based on Associative Classification Data Mining” (Chiew et al., 2019) with 89 citations and the same number of links. Both studies utilize ML for developing anti-phishing systems.

In the second cluster, the most cited article is “CANTINA+: A Feature-Rich Machine Learning Framework for Detecting Phishing Websites” (Xiang et al., 2011) with 135 citations and 33 links, followed by “Cantina: A Content-Based Approach to Detecting Phishing Websites” (Zhang et al., 2007) with 109 citations and the same number of links. The authors of these papers developed and subsequently enhanced a layered solution for phishing webpage detection using ML algorithms.

Finally, in the third cluster, the most cited articles are “Phishing detection based Associative Classification data mining” (Abdelhamid et al., 2014) with 71 citations and 32 links and “Predicting Phishing Websites Based on Self-Structuring Neural Network” (Mohammad et al., 2014) with 68 citations and 32 links. In the first article, the authors investigate the applicability of the Multi-label Classifier based Associative Classification method in detecting phishing websites, highlighting both its performance compared to other intelligent algorithms and its ability to generate new knowledge in the form of associative rules with high predictive value. In the second article, the authors propose an intelligent model for predicting phishing attacks based on self-structuring neural networks.

### 4.3 Thematic analysis and evolution

Thematic analysis and evolution are important bibliometric approaches used to track how research topics emerge, develop, and evolve over time. By analysing keyword co-occurrence networks and the evolution of concepts, valuable insights are gained into shifts in research focus, the emergence of new subfields, and the continuity of key themes over time.

The thematic analysis highlights the topics associated with the use of AI in phishing detection. Topic modeling is a suite of content analysis methods that originates from ML (Blei, 2012). The intellectual structure of the topic was defined based on author keyword co-occurrence analysis and visualized using a strategic map created with Bibliometrix package in R language. Keywords reflect the main topics of a research domain. Analysing authors’ keyword co-occurrence helps identify thematic structures and conceptual relationships between topics. Keywords frequency helps to identify dominant research themes and co-occurrence strength reflects the relationship between these themes. We identified 10 clusters generated based on these keywords by applying the Walktrap algorithm with a Min Cluster Frequency (per thousand docs) set to 10, Number of Words set to 250, Number of Labels to 3 and Label size to 0.3. The Walktrap algorithm is a community detection method used to identify groups of nodes or communities within a network. A random walk starts at a random node and moves to one of its neighbors at each step. Node within a community tends to be more tightly connected, increasing the likelihood that a random walk will remain within that community rather than transitioning to a less connection region of the network (Van Poucke et al., 2018). This algorithm can be successfully used in the thematic analysis of a research field. Min Cluster Frequency (per thousand docs) sets a minimum threshold for the frequency of a theme within the dataset. We set the threshold at 10 to ensure that clusters represent recurring and meaningful topics rather than isolated occurrences, allowing us to include relevant themes while eliminating rare or insignificant ones. The word limit was set at 250 to provide the clustering algorithm with a sufficiently rich vocabulary for meaningful topic differentiation. Choosing three labels per cluster helps maintain interpretability by concisely summarizing the main themes, while the label size was adjusted to enhance readability in visualizations without overwhelming the graphical representation.

For cluster generation, we excluded expressions explicitly containing the terms “phishing” and “AI” (e.g., phishing, phishing detection, phishing attacks, phishing website detection, artificial intelligence, AI, etc.). Additionally, we compiled a list of synonyms to standardize related terms (e.g., blacklist, blacklisting, blacklists; blockchain, blockchains; bot, botnet, botnet applications, botnet detections; deep neural network, deep neural network (dl), deep neural network (dnn), deep neural networks; machine-learning, machine learning, machine learning algorithms, machine learning classifiers, machine learning models, machine learning techniques; malicious, malicious url, malicious url detection, malicious urls, malicious website, malicious websites; malware, malware detection, malware analysis, etc.). This approach ensures a more nuanced analysis by focusing on secondary themes and related concepts. Based on these hyperparameters and restrictions, the application creates ten clusters. Table 6 presents these clusters and their corresponding indicators, including Callon’s centrality and density, rank centrality and density, and cluster frequency.

**Table 6:** Clusters resulted from thematic analysis.

Cluster	Callon centrality	Callon density	Rank centrality	Rank density	Cluster frequency
Web security	0.01	5.88	2	5	17
Classifier	0.06	6.59	8	7	91
Bert	0.02	7.69	3	13	13
Fraud	0.16	7.37	13	12	81
Anomaly detection	0.08	6.90	9	8	52
Detection	0.09	6.19	10	6	53
Machine-learning	0.63	4.84	15	2	1438
Convolution neural network	0.05	5.47	7	3	60
Security	0.15	7.21	12	10	159
LSTM	0.04	7.26	6	11	31
Social engineering	0.39	8.44	14	14	187
Blockchains	0.11	4.20	11	1	61
XGBoost	0.00	10.00	1	15	10
Decision tree	0.02	5.56	4	4	18
Large language models	0.02	7.14	5	9	14

**Figure 5:**
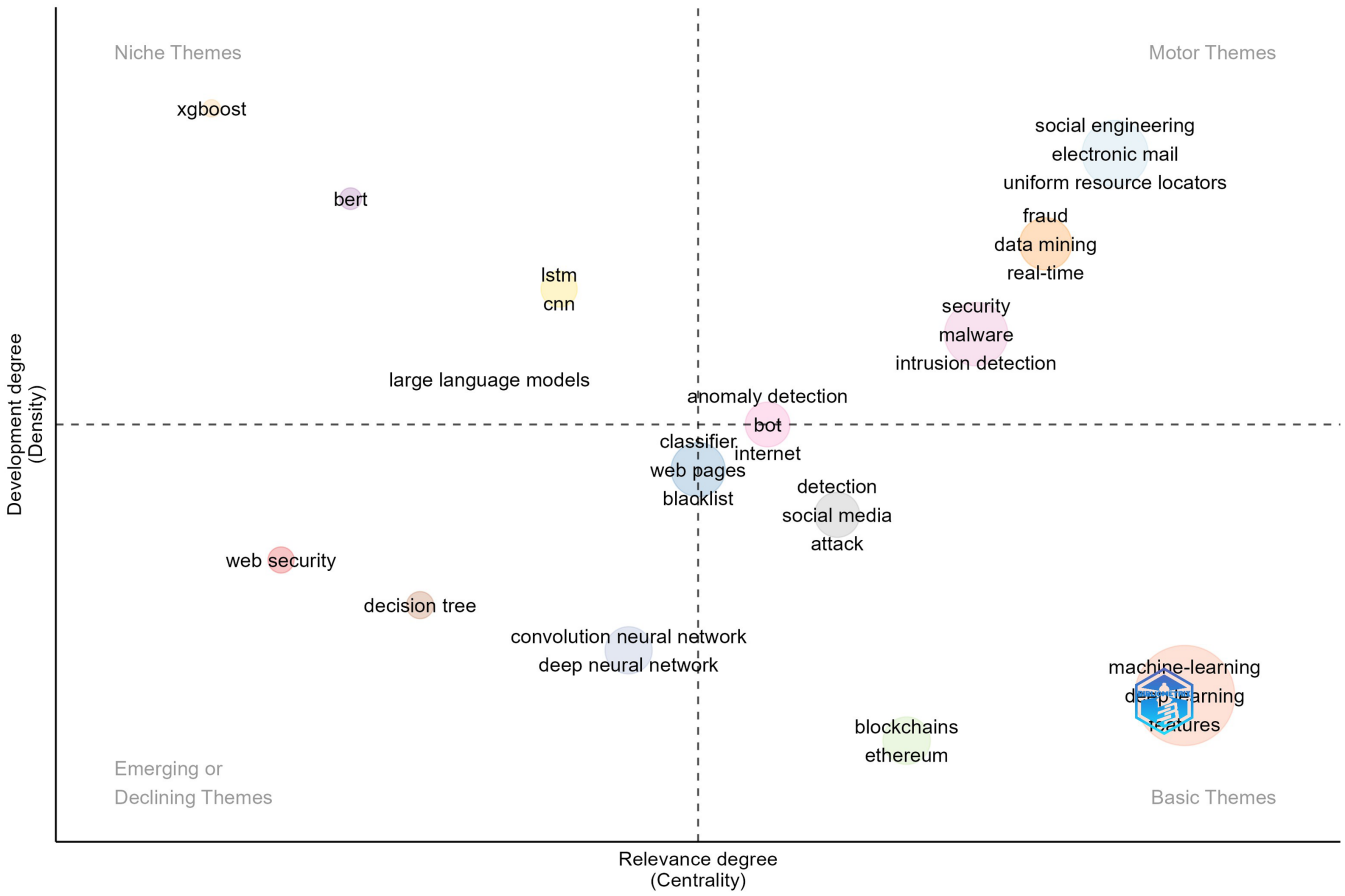
Thematic map.

Thematic clusters are positioned in a two-dimensional space, allowing for the identification of core and peripheral themes. Centrality reflects the relevance of a theme, identifying dominant and emerging themes. Density measures the internal cohesion of a theme and indicates its level of development. Callon centrality measures the interaction between networks. A high centrality signals that a node has many connections with other nodes, reflecting its potential influence in spreading ideas and information within the network. In the analysis context, *ML*, *social engineering*, and *fraud* are the topics with the highest centrality, playing a crucial role in the research within this field. Callon density measures the cohesion between nodes. A high density reflects a strong connection between themes and a coherent structure. In this case, eXtreme Gradient Boosting (XGBoost) has the highest Callon density value. Rank centrality and rank density provide information on the relative importance of nodes within a cluster, while cluster frequency represents the number of appearances in the dataset. A higher frequency indicates themes that appear more frequently in the dataset articles. Here, ML has a significantly higher frequency compared to other clusters. Table 6 is the basis for Figure 5. Each bubble represents a network cluster. The words within each cluster that define its name are those with the highest occurrence, and the size of the bubble is proportional to the frequency of those words. The centrality and density of the cluster, according to Callon’s measures, are reflected in the position of the bubble. The thematic map provides a structured visualization of research topics, categorizing them based on their relevance and development within a field. This helps in understanding the intellectual structure of a research domain, identifying well-established themes, and detecting emerging trends.

In the first quadrant, motor themes are characterized by a high degree of relevance and development, being essential for organizing the study topic. In this context, these themes are associated with: *social engineering* (Innab et al., 2024; Perera and Grob, 2024), *network security*, particularly in the context of the development of cloud computing (Dawood et al., 2023), the use of *data mining* (Alshahrani et al., 2022; Bejandi et al., 2022) and *combating phishing fraud*, a concern driven by the significant number of financial frauds in recent years (Iscan et al., 2023). Other topics included in this quadrant are general concepts or tools related to phishing attacks such as *electronic email*, *uniform resource locator*, *real-time*, *malware* and *intrusion detection*.

In the second quadrant, niche themes exhibit a high level of development but a lower degree of relevance. For the analyzed dataset, this category includes topics encompasses various technologies used in cyber-attack detection and mitigation, including *XGBoost* (Agagu et al., 2024; Gualberto et al., 2020; Omari, 2023), *large language models* (Heiding et al., 2024; Trad and Chehab, 2024), *Bidirectional Encoder Representations from Transformers (BERT)* (Thapa et al., 2023) and *Long Short-Term Memory (LSTM)* (Gopali et al., 2024; Orunsolu et al., 2022). Their position in this quadrant reflects that while these topics are well-developed, they have not yet attained central importance within the broader scientific field.

In the third quadrant, emerging or declining themes exhibit low density and centrality, indicating that they are in a developing stage. In the analyzed context, the use of *decision tree*, *convolution neural network* and *deep neural network* are less developed and have low centrality. It also includes a more generic topic related to phishing attack as *web security*. However, the theme could become more prominent due to the potential of using ML in identifying cyber-security attacks such as smishing (Jain and Gupta, 2018a).

The last quadrant, basic themes, includes topics with high relevance but low development, typically serving as foundational elements for understanding the field. In the analyzed dataset, this quadrant encompasses: *ML* and *DL*, *blockchain, mitigation of phishing attacks on the Ethereum platform*, the second-largest blockchain platform (Li et al., 2022), the use of *social media* for phishing attacks (Aun et al., 2023; Khan and Unhelkar, 2024). These themes are considered basic due to their foundational role in the study area, despite their current lower level of development.

Some topics lie at the intersection of different quadrants. The use of *bots for detecting anomalies* is positioned on the boundary between the first and fourth quadrants. This suggests a subject that is central to the field, with high relevance but an intermediate level of development. Future research may lead to the consolidation of work on the use of bots in detecting phishing attacks, or it may remain only a reference point for other future research directions. Similarly, concerns regarding the creation of *blacklists* and the use of *ML and DL classifiers* are located at the intersection of the penultimate two quadrants. This positioning suggests that these directions are recognized as relevant and connected to the core themes of the field but are still at a relatively early stage of development.

The thematic evolution of the research field reflects the dynamic changes in its core topics over time, revealing trends, emerging areas, and the persistence or decline of specific themes. This analysis provides valuable insights highlighting key trends and shifts in focus. Throughout the analyzed period, the theme associated with the use of AI in phishing detection has evolved and transformed due to both the expansion of the phenomenon and technological advancements. This analysis was undertaken using Bibliometrix package. A longitudinal analysis is conducted by segmenting data into time periods to analyse thematic evolution. We divided the analyzed period into three-time frames of different lengths to account for variations in the field’s evolution. The first period covers 10 years (2005–2015), as research in this area was more limited, and topics were less diverse. The last two periods were segmented into two intervals (2016–2020 and 2021–2025), reflecting the increasing diversification of topics related to the use of AI for phishing detection. Table 7 provides details on these transformations based on authors’ keywords. The same list of excluded terms and synonyms used in the thematic analysis was applied in the case of thematic evolution. Among the hyperparameters, only the Min Cluster Frequency (per thousand docs) value was modified to 5. The remaining parameters—Number of Words, Weight Index, Min Weight Index, Label, Number of Labels (for each cluster), and Clustering Algorithm—remained unchanged. Specifically, Number of Words was set to 250, Min Weight Index was set to 0.1, Label was set to 0.3, Number of Labels (for each cluster) was set to 3, and for the Clustering Algorithm, we selected Walktrap. The Weight Index chosen for thematic evolution was the Inclusion Index weighted by Word Occurrences. To establish the parameters, we applied the same reasoning as in the thematic analysis.

**Table 7:** Thematic evolution.

From	To	Weighted inclusion index	Inclusion index	Occurrences	Stability index
Anomaly detection—2005–2015	Anomaly detection—2016–2020	1.00	1.00	5	0.11
Anomaly detection—2005–2015	Computer crime—2016–2020	0.17	0.17	2	0.07
Anomaly detection—2005–2015	Deep learning—2016–2020	0.10	0.11	2	0.04
Boosting—2005–2015	Machine-learning—2016–2020	0.33	0.33	2	0.05
Decision tree—2005–2015	Random forest—2016–2020	1.00	1.00	2	0.50
Machine-learning—2005–2015	Computer security–2016–2020	1.00	1.00	4	0.08
Machine-learning—2005–2015	Machine-learning—2016–2020	0.70	0.08	23	0.04
Machine-learning—2005–2015	Social engineering—2016–2020	0.13	0.33	3	0.07
Accuracy—2016–2020	Security—2021–2025	0.67	0.50	4	0.06
Anomaly detection—2016–2020	Anomaly detection—2021–2025	1.00	1.00	2	0.20
Bot—2016–2020	Bot—2021–2025	1.00	1.00	8	1.00
Computer crime—2016–2020	Convolution neural network—2021–2025	0.17	0.25	2	0.11
Computer crime—2016–2020	Deep neural network—2021–2025	0.17	0.50	2	0.14
Computer security—2016–2020	Security—2021–2025	1.00	1.00	2	0.06
Convolution neural network—2016–2020	Convolution neural network—2021–2025	0.64	0.33	9	0.17
Cyberattack—2016–2020	Machine-learning—2021–2025	1.00	1.00	3	0.03
Cybercrime—2016–2020	Machine-learning—2021–2025	1.00	1.00	5	0.03
Deep learning—2016–2020	Anomaly detection—2021–2025	0.14	0.20	7	0.04
Deep learning—2016–2020	LSTM—2021–2025	0.09	0.33	3	0.05
Deep learning—2016–2020	Machine-learning—2021–2025	0.73	0.05	32	0.02
Deep learning—2016–2020	Security—2021–2025	0.02	0.06	2	0.03
Detection—2016–2020	Machine-learning—2021–2025	1.00	1.00	5	0.03
Ensemble—2016–2020	Machine-learning—2021–2025	0.57	0.50	4	0.03
LSTM—2016–2020	LSTM—2021–2025	1.00	1.00	2	0.33
Machine-learning—2016–2020	Blockchains—2021–2025	0.07	0.25	6	0.05
Machine-learning—2016–2020	Machine-learning—2021–2025	0.84	0.06	120	0.02
Machine-learning—2016–2020	Security—2021–2025	0.05	0.06	9	0.03
Mobile phishing—2016–2020	Machine-learning—2021–2025	0.18	0.25	2	0.03
Random forest—2016–2020	Convolution neural network—2021–2025	0.46	0.50	6	0.20
Random forest—2016–2020	Machine-learning—2021–2025	0.54	0.50	7	0.03
Social engineering—2016–2020	Security—2021–2025	0.74	0.33	14	0.06
Social media—2016–2020	Machine-learning—2021–2025	0.71	0.50	5	0.03
Web security—2016–2020	Machine-learning—2021–2025	0.78	0.50	7	0.03
XGBoost—2016–2020	Machine-learning—2021–2025	1.00	1.00	3	0.03

The metrics used to quantify the transition or stability of the analyzed themes are weighted inclusion index, inclusion index, number of occurrences and stability index. These metrics offer an overview of the trends over time and the importance of specific topics based on the articles in the dataset. Weighted inclusion index and inclusion index are normalized metrics of relevance and overlap of themes, with values ranging from 0 to 1. A value of 1 indicates maximum overlap or relevance. Occurrences reflect the number of documents supporting the transition of themes. Stability index indicates the stability of a research theme between consecutive periods. A value closer to 1 indicates a higher number of studies supporting the transition of the theme. These metrics together provide a comprehensive view of how themes related to AI in phishing detection have developed and shifted over time, reflecting their growing or diminishing importance in the research landscape.

Each line in the table represents a change or continuation in research on a specific topic. A perfect transition is indicated by a value of 1 for the weighted inclusion index, inclusion index, and stability index. The weighted inclusion index and inclusion index both have a value of 1 for the following themes: Decision Tree to Random Forest—a perfectly expected transition as Random Forest is an extension of Decision Tree (2005–2015 to 2016–2020); cyberattack, cybercrime to ML (2016–2020 to 2021–2025), and XGBoost to ML (2016–2020 to 2021–2025). This value indicates a maximum overlap or relevance between the mentioned themes.

Research in the fields of bots and LSTM continuity (2016–2020 to 2021–2025) reflects a consistent interest. However, LSTM has a smaller number of occurrences (2) and lower stability (0.33), whereas bots is a topic with a higher number of occurrences (8) and with string stability (1). This indicates that while bots maintain a strong presence with extensive research, the topic of LSTM has a broader and less stable presence. Furthermore, ML (2005–2015 to 2016–2021 and 2016–2020 to 2021–2025) shows continuity, reflecting the stability of the topic over the entire period analyzed, with a high weighted inclusion index in both cases. The lower inclusion index indicates a constant but slightly reduced interest, which may suggest both progress and saturation in the research efforts related to ML for anti-phishing. On the other hand, the very high number of occurrences of the concept (120) in the latter period might be an indicator of significant progress in the field.

**Figure 6:**
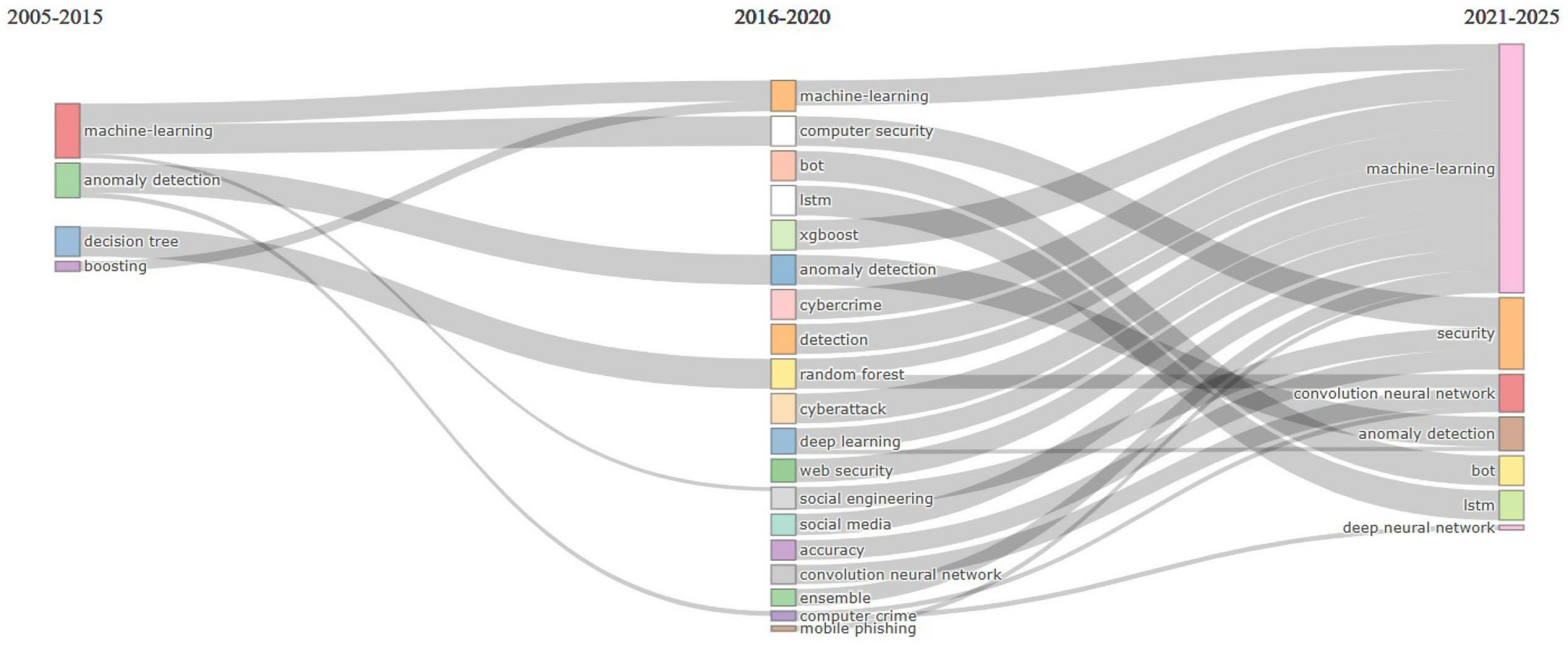
Thematic evolution based on authors’ keywords.

The analysis period was divided into three slices: from 2005 to 2015, from 2016 to 2020, and from 2021 to 2025 (Figure 6). This decision was influenced by the evolution of the number of published works on the analyzed topic across these periods and by the diversity of topics. In the first slice, the number of published papers was very small, with authors’ concerns focused on two research directions: ML, anomaly detection, boosting and decision trees. In the subsequent period, the number of published papers on the subject increased significantly, showing an upward trend until 2022 and from 2023 to 2024. Themes that appeared at least ten times per thousand documents (Min Cluster Frequency (per thousand docs)) were considered relevant for inclusion in the graphic. We set the threshold at 10 to ensure that clusters represent recurring and meaningful topics, not isolated ones.

In the second period, initial themes evolved and led to the emergence of new topics. ML was carried over into subsequent periods in its original form but also led to a new direction, focusing on computer security for phishing identification. Decision Tree evolved into Random Forest and then focused on ML and CNN in the period 2021–2025. During the 2016–2020 period, new themes related to AI in phishing detection emerged, including bot, LSTM, XGBoost, CNNs, cyberattack, cybercrime, deep learning, web security, social engineering and social media, etc. The Random Forest topic from 2016 to 2020 expanded into two topics: ML and CNNs. Themes such as Random Forest, ML, deep learning, cybercrime, accuracy detection, and cyberattack from 2016 to 2020 merged and reformed into the theme of ML for 2021–2025.

## 5 Discussion

### 5.1 RQ1. How has the publication landscape in AI for phishing detection research evolved over time?

Phishing attacks are widespread across the globe, and the methods to counter them are also of global interest (Mutluturk and Metin, 2023). The results presented in the previous section highlight a *significant growth* and *specialization in research* on the use of AI for detecting and mitigating phishing attacks, particularly in recent years. This trend can be attributed to a combination of factors. On one hand, the frequency of phishing attacks has increased markedly, while on the other hand, AI has become substantially more advanced, continuously enhancing its ability to understand complex behaviors, detect patterns within large datasets, and adapt to identify progressively sophisticated phishing techniques. The authors of the analyzed papers focused on subdomains of AI, such as ML, DL, and NLP, to identify the most effective algorithms and methods to improve the results obtained in phishing prevention and detection.

**Figure 7:**
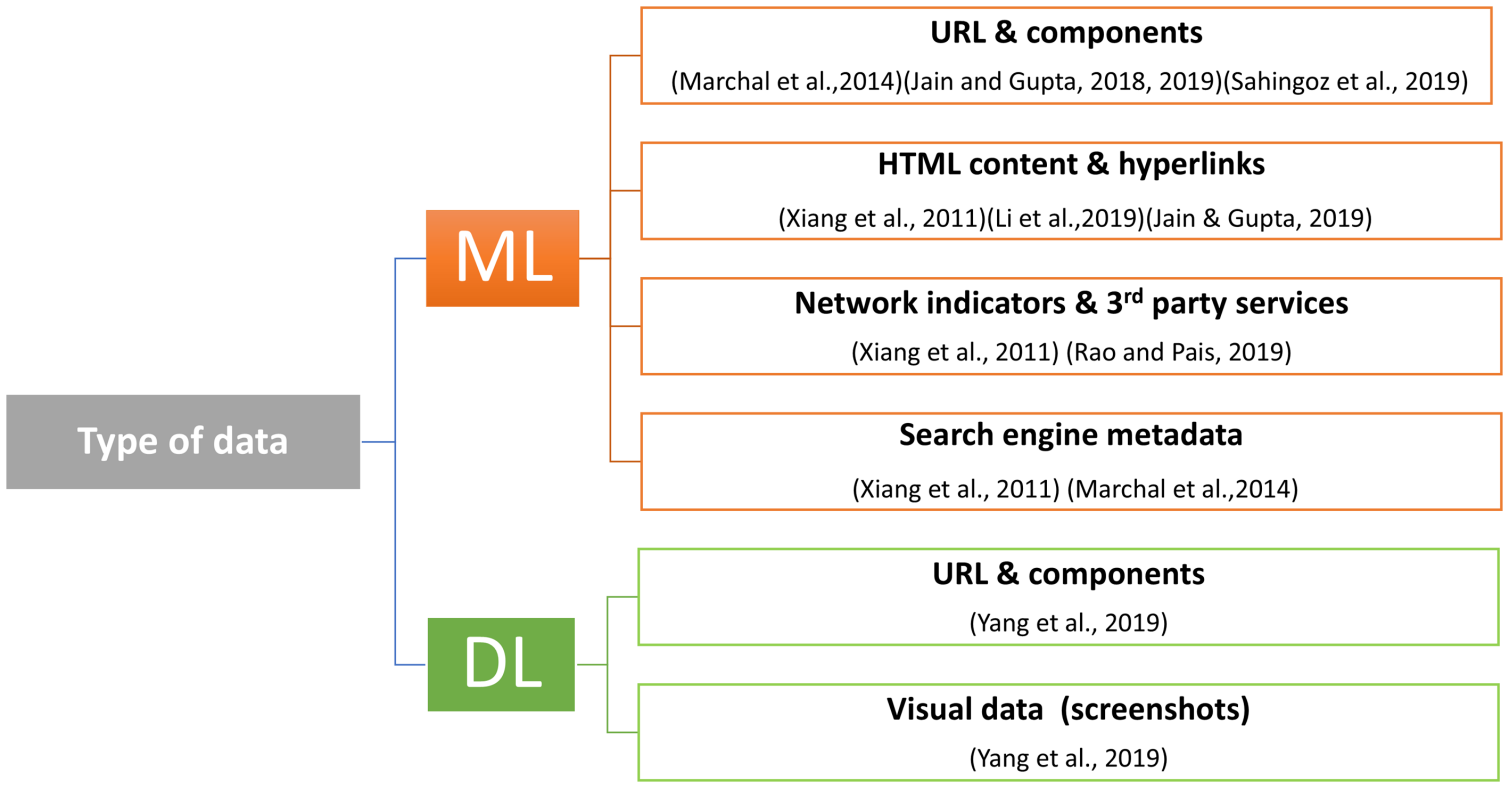
Types of data used in phishing detection.

**Figure 8:**
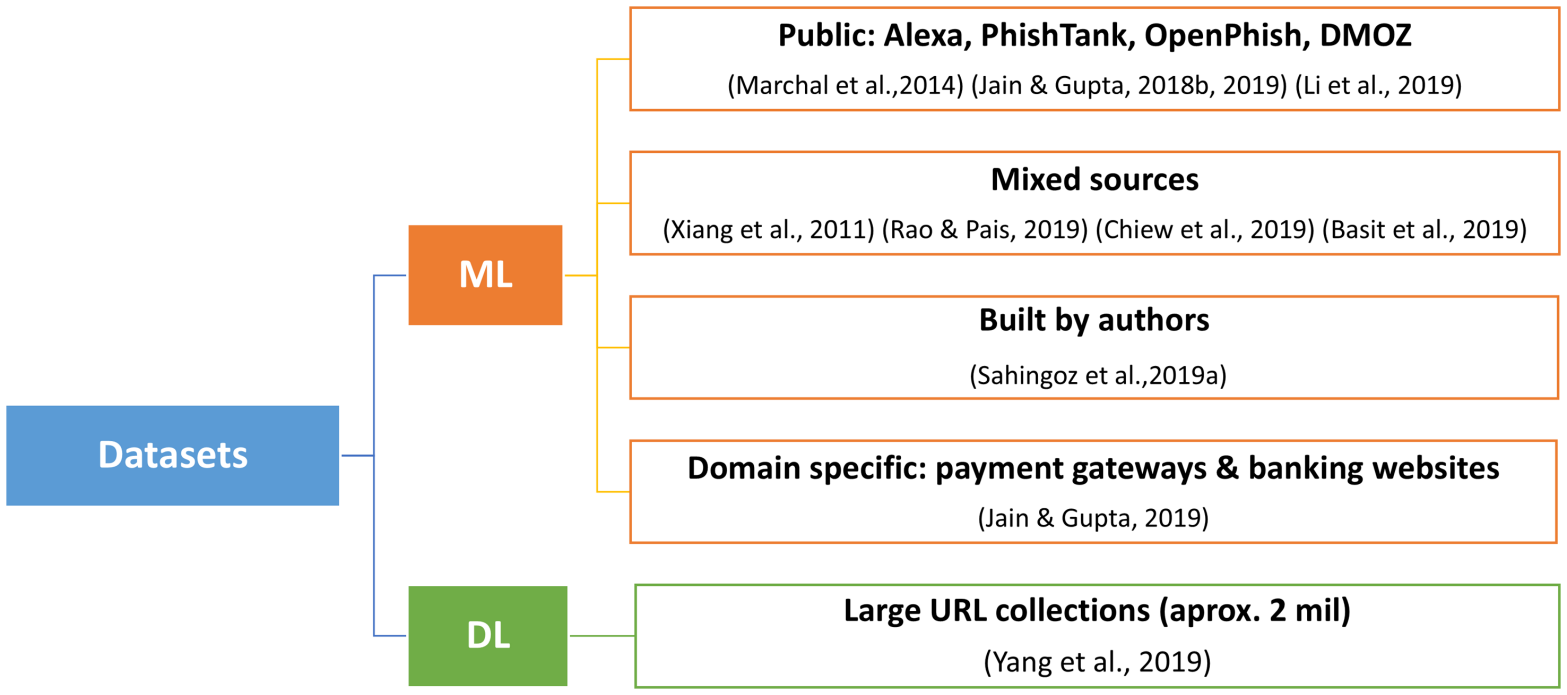
Datasets used in phishing detection.

The top 10 most cited studies identified through our bibliometric analysis focus on phishing detection using ML methods and, more recently, DL techniques. The types of data analyzed by these algorithms include URLs and their components, HTML content and hyperlinks, JavaScript behavior, network indicators and third-party services, as well as metadata from search engines, among others. A taxonomy of the types of data used by the most cited articles is presented in Figure 7, and the classification of the used datasets is visible in Figure 8.

**Figure 9:**
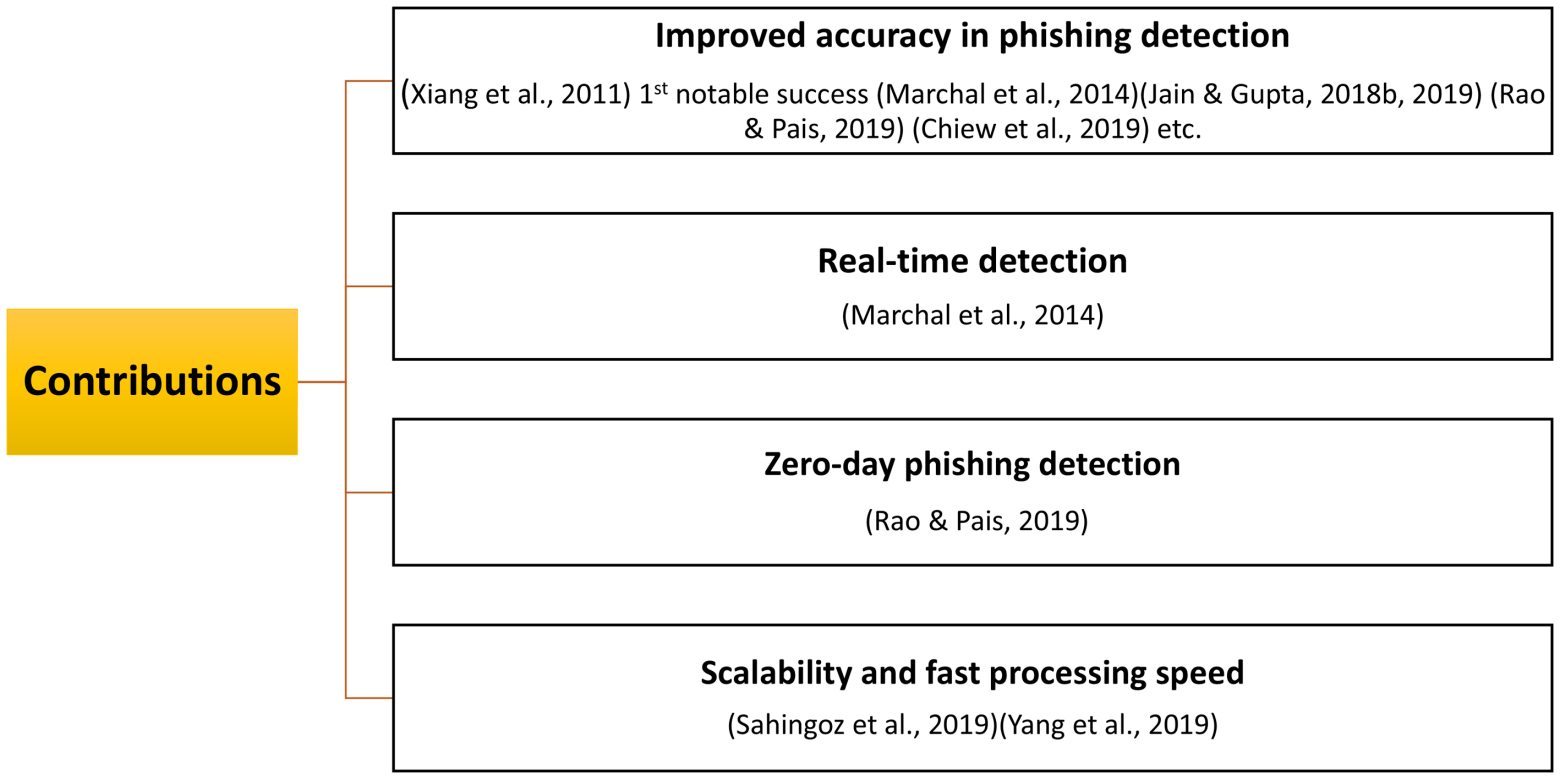
Main contributions of the most cited studies.

The main objectives pursued by the authors are to identify discriminative features between legitimate and phishing websites, develop ML/DL models for websites’ classification, reduce false alarm rates and response times, and design scalable real-time solutions. The results reported by these authors are presented synthetically in Table 8 and Figure 9.

**Table 8:** A synthesis of the work of the most cited 10 articles in our dataset.

Title	Method/model	Used data	Algorithms/techniques	Main findings
*Toward Detection of Phishing Websites on Client-Side Using Machine Learning Based Approach* (Jain and Gupta, 2018b)	ML on multiple datasets	Phishtank, OpenPhish, Alexa, payment gateways, banks	RF, SVM, Neural Nets, Logistic Regression, Naive Bayes	Improved accuracy using client-side data extraction
*Detection of Phishing Websites Using an Efficient Feature-Based Machine Learning Framework* (Rao and Pais, 2019)	Feature extraction from URL + source code + 3rd parties	Diverse data sets	8 ML algorithms	Better than CANTINA/CANTINA+, detects zero-day phishing
*Phishing Website Detection Based on Multidimensional Features Driven by Deep Learning* (Yang et al., 2019)	CNN for phishing detection	~2M URLs (1,021,758 phishing + 989,021 legitimate)	CNN	High performance and fast processing speed
*Machine Learning Based Phishing Detection from URLs* (Sahingoz et al., 2019)	Custom dataset + NLP	73,575 URLs (36,400 legitimate, 37,175 phishing)	DT, AdaBoost, K-star, kNN, RF, SMO, Naive Bayes	Scalable, real-time, detects new phishing attempts
*PhishStorm: Detecting Phishing with Streaming Analytics* (Marchal et al., 2014)	PhishStorm – real-time detection	PhishTank, DMOZ: URLs + search engine queries	Classical ML on URL components	94.91% accuracy, 1.44% false positives (FP)
*A Machine Learning Based Approach for Phishing Detection Using Hyperlinks Information* (Jain and Gupta, 2019)	HTML hyperlinks analysis	PhishTank, OpenPhish, Alexa: Hyperlinks from source code	Logistic Regression + 12 hyperlink features	Achieved 98.4% accuracy, language-independent
*A New Hybrid Ensemble Feature Selection Framework for Machine Learning-Based Phishing Detection System* (Chiew et al., 2019)	HEFS + CDF-g for optimal feature selection	Multiple sources	Ensemble framework	Improves accuracy through optimal feature selection
*A Stacking Model Using URL and HTML Features for Phishing Webpage Detection* (Li et al., 2019)	Stacking model on URL + HTML features	Phishtank (2k webpages) + Alexa (49,947 webpages)	Combined SVM, NN, DT, RF	High accuracy, stacking outperforms individual models
*CANTINA+: A Feature-Rich Machine Learning Framework for Detecting Phishing Web Sites* (Xiang et al., 2011)	Extraction of 15 high-level webpage characteristics from URLs, HTML DOM, 3rd party services, search engines	Diverse Web resources	SVM, Logistic Regression, Bayesian Network, J48, Random Forest, AdaBoost	Good TP/FP rate, competitive solution
*A Comprehensive Survey of AI-Enabled Phishing Attacks Detection Techniques* (Basit et al., 2021)	Review on phishing	Diverse datasets	RF, SVM, kNN	ML and DL have up to 99% accuracy, much better than heuristics and data mining approaches

**Figure 10:**
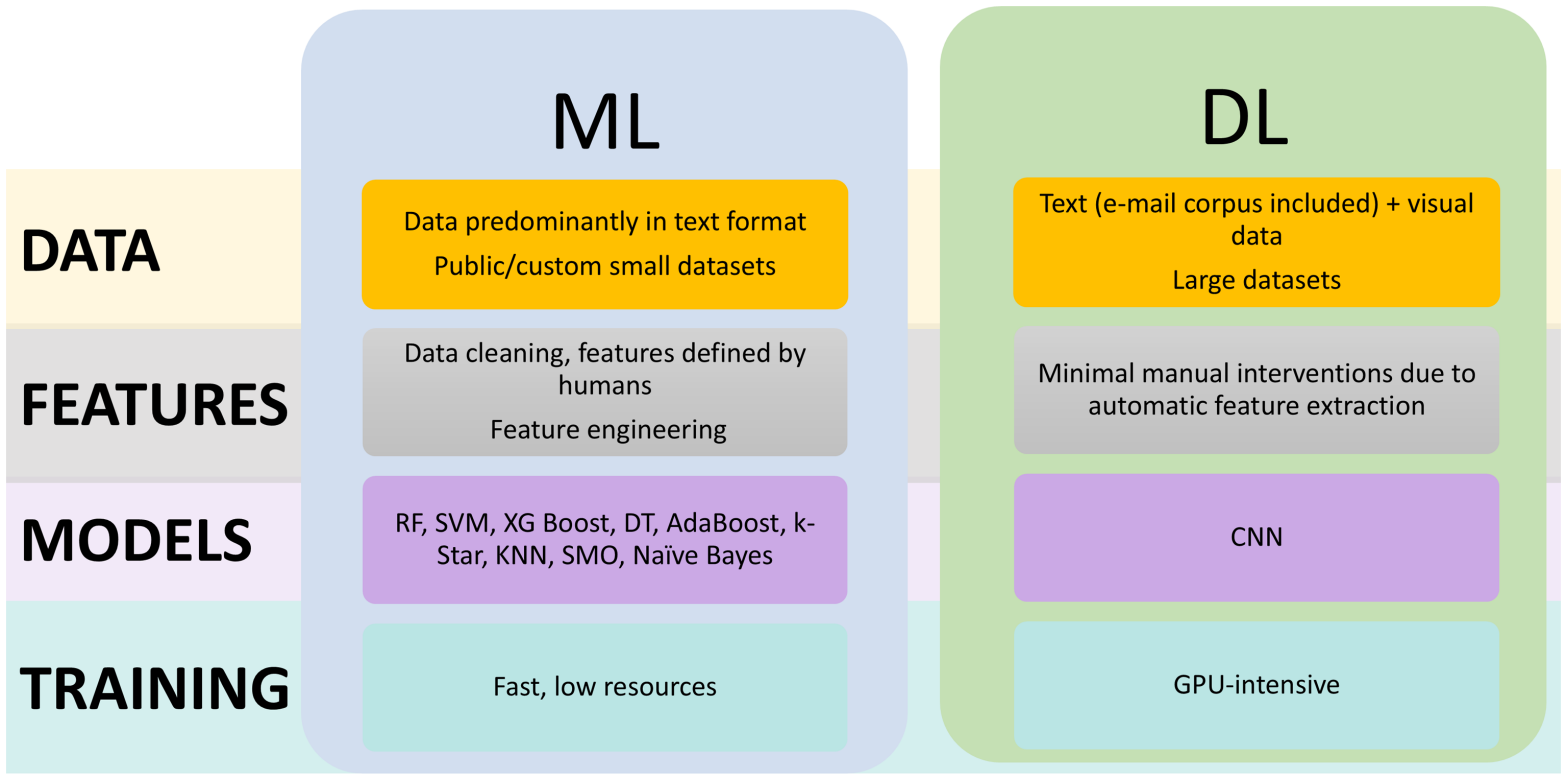
ML versus DL. Differences in phishing detection process.

The key-trends extracted after an in-depth content analysis of the top 10 most cited articles ranked by Local Citation Rate (LCR)—a metric reflecting their relevance within the domain – are:

1. *Growing adoption of ML methods*. ML techniques are increasingly used for phishing detection, with frequently employed algorithms including Random Forest, Support Vector Machines, Neural Networks, Logistic Regression, Naive Bayes, k-Nearest Neighbors, Decision Trees, and AdaBoost. Using an extended number of features of different types (e.g. URLs’ configurations, source code, 3rd party services data) and being applied on large and diverse datasets (Alexa, PhishTank, OpenPhish, payment gateways data, or own data collected/generated by authors), these approaches achieve high detection rates compared with traditional methods: for instance Basit et al. (2021), while (Wang et al., 2019) calculated a 97% accuracy for an algorithm based on CNN.
2. *Shift from classical ML to DL*. While classical ML algorithms dominated research until 2018 and achieved strong performance, they required feature sets curated by people and exhibited limitations in detecting previously unseen phishing attacks (e.g., tiny URLs, BiB – false authentication windows). After 2018, DL techniques—such as CNNs, DNNs, and stacking-based architectures—gained significant traction, offering higher accuracy, better scalability, and improved generalization. In particular, the adoption of CNNs has grown due to their ability to capture local correlations in data, especially in Big Data environments (Yang et al., 2019). To mitigate long training times associated with DL (Sahingoz et al., 2019), recommend leveraging parallel processing techniques. Figure 10 presents the main differences between ML and DL in phishing detection process.
3. *Increasing importance of feature selection and engineering*. Feature engineering plays a critical role in enhancing detection performance. For example, Chiew et al. (2019) introduce the Hybrid Ensemble Feature Selection (HEFS) framework and the CDF-g algorithm for optimal feature selection. Modern approaches typically combine URL-based features (e.g., length, entropy, number of subdomains), HTML-based features (e.g., suspicious links, login forms), and third-party data (e.g., Alexa rank, domain reputation).
4. *Hybrid models and classifier stacking*. The analyzed studies demonstrate that combining ML and DL models with stacking-based approaches (Li et al., 2019) significantly improves detection performance. Furthermore, integrating feature selection as a preprocessing step enhances accuracy by focusing on the most relevant attributes within the dataset.
5. *Real-time phishing detection*. Real-time detection systems are considered a priority. For instance, PhishStorm (Marchal et al., 2014) introduces an automated, Big Data-supported URL analysis framework capable of achieving 94.91% accuracy with a 1.44% false positive rate.
6. *Faster, scalable, and language-independent systems*. Modern solutions are evolving toward client-side architectures that are fast, lightweight, and less dependent on third-party databases. This independence enhances scalability and robustness. Additionally, continuous model retraining on large, up-to-date datasets is increasingly adopted to maintain high adaptability against evolving phishing strategies.

Overall, the field is witnessing a paradigm shift—from traditional ML-based approaches toward hybrid, DL-driven, real-time detection systems that integrate advanced feature engineering and scalable architecture. These developments position AI-powered solutions as the cornerstone of next-generation phishing defense mechanisms.

### 5.2 RQ2. What are the core thematic clusters within the AI-based phishing detection research field, and how do these clusters interact and evolve over time?

The field is anchored around social engineering, network security, and ML/DL-based detection, while advanced methods like LLMs, BERT, CNNs, and blockchain are gaining traction. Future research is likely to consolidate work on bot-based anomaly detection and expand AI techniques for emerging phishing vectors such as smishing and social media attacks.

**Figure 11:**
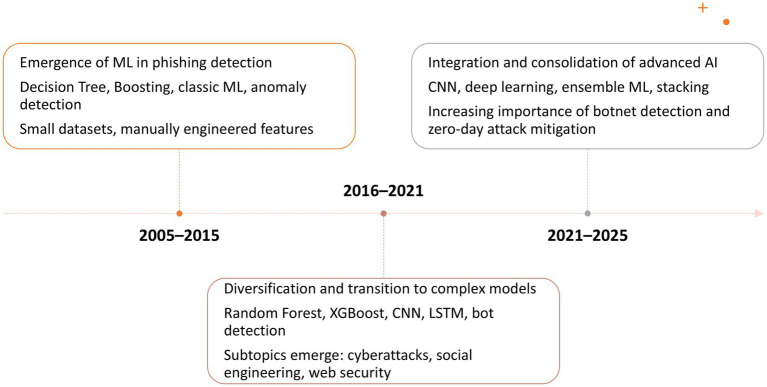
Main trends in time.

Research on AI-based phishing detection has evolved rapidly over the past two decades. In its initial stage (2005–2015), studies were limited and relied on basic ML techniques applied to URL and webpage classification using small, manually engineered datasets. The expansion phase (2016–2020) brought a surge in publications and the adoption of more advanced ML and DL methods, including Random Forests, CNNs, LSTMs, and XGBoost, alongside emerging themes such as bot detection, social engineering, and web security. In the consolidation phase (2021–2025), research has shifted toward integrated ML–DL frameworks, with CNN-based architectures dominating large-scale phishing detection, ensemble methods becoming standard, and bot detection maturing as a stable area. While ML remains central, there is a clear trend toward hybrid, AI-driven solutions that enhance accuracy, scalability, and zero-day detection capabilities (Figure 11).

Regarding the thematic evolution of the field, the analysis conducted using Bibliometrix reveals a gradual shift from simple approaches to increasingly complex methodologies based on ML and DL, accompanied by the emergence of new research directions:

- ML remains the foundation of phishing detection but is increasingly complemented by DL and hybrid models;
- CNNs are becoming the de facto standard for handling large-scale datasets and detecting complex patterns;
- XGBoost and Random Forest remain core algorithms but are now frequently integrated into ensemble-based detection systems;
- Bot detection and social media analytics are gaining prominence in the context of large-scale phishing campaigns;
- Scalability, real-time detection, and the ability to identify zero-day phishing attacks have become critical priorities;
- As classical ML approaches reach a saturation point, the focus is shifting toward integrating advanced AI techniques to achieve higher accuracy and greater adaptability.

### 5.3 RQ3. How are the AI technologies identified in the study utilized within organizations?

**Figure 12:**
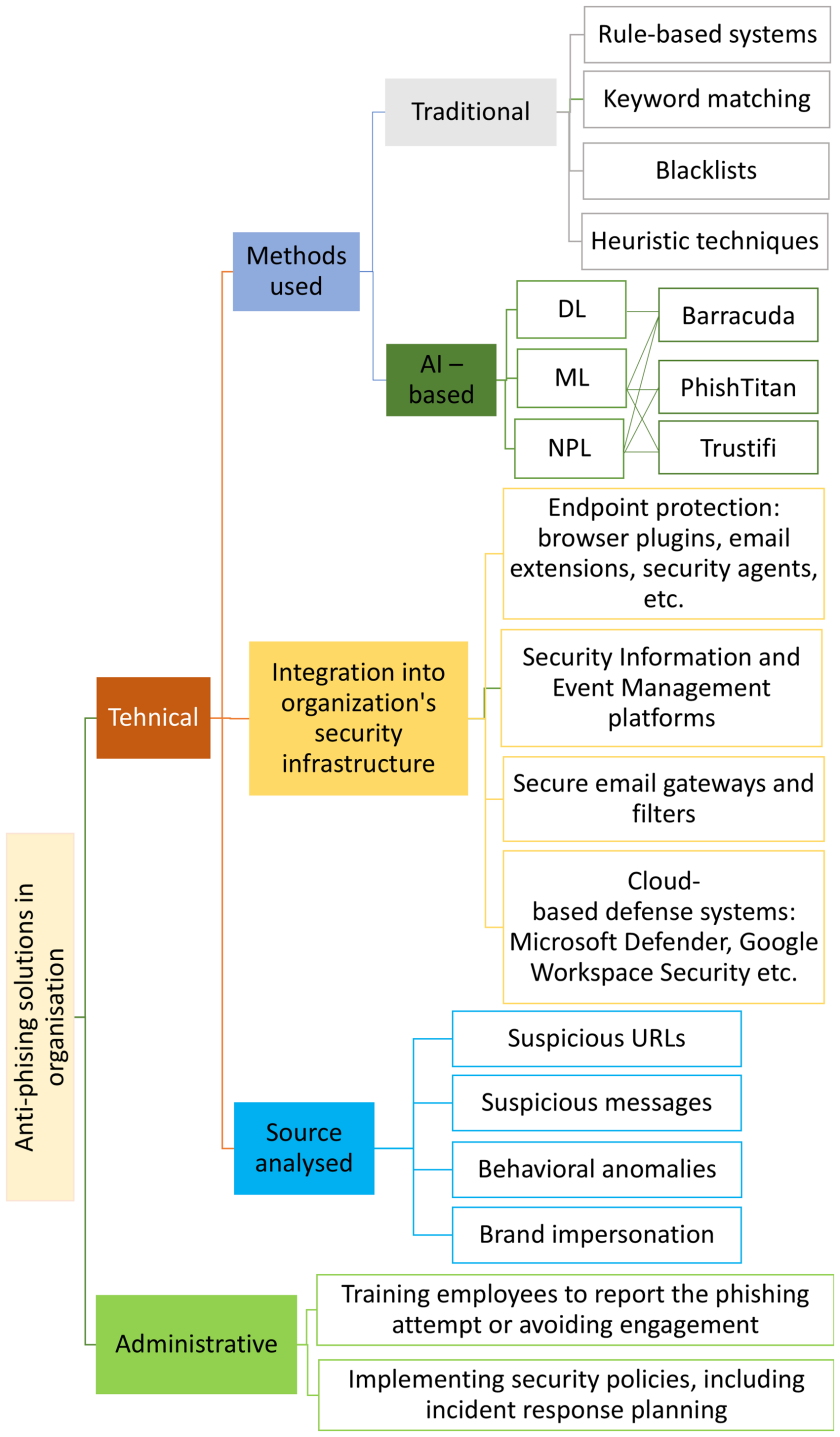
Practical implications—a taxonomy.

An important aspect highlighted by recent research is that the most effective solutions for protecting potential phishing victims need to be implemented at the organizational level through the application of technology security governance, following strict taxonomy classification (Pejić-Bach et al., 2023). These observations reiterate the potential of AI, given the significant resources required for the implementation of these technologies. Figure 12 presents a taxonomy of anti-phishing solutions within organizations both based on traditional methods and based on AI.

Developing effective systems for phishing attack detection remains a persistent challenge for **cybersecurity experts**. While traditional methods use mostly rule-based systems, keyword matching, blacklists, and heuristic techniques, current approaches predominantly leverage ML and DL algorithms; however, these techniques often exhibit high false-positive rates and demand substantial computational resources (Basit et al., 2021) and access to relevant and updated large datasets. The significance of AI in detecting phishing attacks is primarily attributed to the capacity of ML and DL models to learn from new data and enhance detection accuracy over time. The current analysis of the most relevant studies identified through bibliometric tools highlights a sustained focus on improving the algorithms employed, particularly in response to challenges such as the shift of phishing attacks to mobile platforms, the targeting of multilingual websites, and the evolving nature of phishing tactics. AI’s ability to integrate diverse data sources (e.g., images) and adapt to new attack patterns positions it as an important tool in addressing the existing gap in detection systems, enhancing both their robustness and adaptability.

**The interest of cybersecurity companies in integrating AI into phishing detection** mechanisms is increasingly evident. Besides the AI-powered anti-phishing techniques used by giants like Microsoft and Google to provide safe browsing for the users, divers measures are adopted by smaller players on the cybersecurity market. For example, Barracuda employs AI to enhance phishing detection by analysing email content in real-time, identifying anomalies, and automating remediation while continuously adapting to emerging threats. The system refines its detection models through ML that assess behavioral deviations, minimizing false positives. Additionally, AI-powered anomaly detection in Barracuda’s Managed XDR (eXtended Detection and Response) establishes security baselines, identifies suspicious activities, and enables proactive threat mitigation across diverse environments (Barracuda, 2024). PhishTitan applies ML algorithms to analyse email content, inspect headers, and identify potential phishing attempts. Curated threat intelligence feeds assist in detecting malicious URLs, while real-time URL analysis and rewriting help mitigate risks (TitanHQ, 2024).

In both cases, integration with Microsoft 365 enables enhanced security measures against phishing threats. Trustifi’s cloud-based security solution employs text-based AI to detect impersonation, spoofing, spear-phishing, and business email compromise while scanning URLs and attachments for malicious content. AI-powered filtering mechanisms ensure inbox hygiene by eliminating spam and graymail, thereby mitigating phishing risks (Trustifi, 2024). Abnormal employs AI and ML through computer vision and NLP to analyse email content, benchmark behaviors, and assess risk in account activity. Multiple AI models, including identity and behavioral mapping, BERT large language models, and risk profiling, are integrated to detect anomalies and correlate threats based on user identity fluctuations, event context, and risk assessment. AI automates email security management by understanding user behavior, remediating attacks, correlating malicious reports, and leveraging conversational AI for real-time security training, while continuously refining detection capabilities with large language models (Shiebler, 2023). On this background, cybersecurity solutions developers can leverage the findings of this study to identify the most effective technologies and algorithms for phishing detection and subsequently integrate them into their own products, provided that sufficiently large datasets are available to support ML and DL models. Moreover, the identification of the most relevant authors, their geographic distribution, and their collaboration patterns presented in this study can facilitate partnerships between academia and industry, accelerating the transfer of theoretical advancements into practical cybersecurity solutions. Additionally, the identification of key topics within author clusters enables organizations to align their research and development strategies with cutting-edge innovations in phishing detection.

The **integration of AI-based solutions for detecting suspicious URLs and messages into an organization’s security infrastructure** can be implemented in various ways. AI-generated phishing alerts can be incorporated as a functionality within enterprise security dashboards and security information and event management (SIEM) systems (PaloAltoNetworks, 2025). AI phishing detection works alongside antivirus software and firewalls in endpoint security solutions (SentinelOne, 2025), and is embedded by services like Microsoft Defender, Google Workspace Security, and third-party cybersecurity platforms in cloud email protection solutions (Nathanson and Yamunan, 2025). These integrations strengthen an organization’s defense against phishing by providing comprehensive monitoring, real-time threat detection, and automated responses across various platforms and devices. Cybersecurity officers can contribute by selecting solutions such as email security gateways, web filters, or behavioral analysis applications, implementing, testing, and comparatively analyzing them in terms of performance and in relation to traditional applications without AI integration. Additionally, they can support the process by collecting user feedback on AI-enhanced anti-phishing software from the systems they manage and reporting it to application developers. Legitimate and malicious emails, URLs accessed by employees, and log data can be collected and shared with dataset creators for training detection algorithms. This crowdsourcing approach aims to mitigate the impact of phishing on companies by increasing both the number and diversity of cases used in algorithm training, enhancing their ability to distinguish between harmful and safe messages.

While AI plays an important role in automated detection, human awareness remains an essential component of phishing mitigation. AI-based phishing detection systems not only neutralize threats but can also educate users about the risks associated with phishing attempts. When a phishing attempt is detected, AI-generated alerts provide users with contextual information about the nature of the threat. These alerts may include explanations of why an email is suspicious, potential consequences of interacting with the message, and recommended actions, such as reporting the phishing attempt or avoiding engagement. Security awareness training platforms leverage AI-generated phishing simulations to test users’ responses to deceptive emails, helping organizations assess employee susceptibility to phishing attacks. Studies have shown that periodic phishing awareness training, combined with real-time AI-generated warnings, significantly reduces user engagement with phishing attempts (SentinelOne, 2025).

Regarding the **formulation of relevant policies**, this study highlights several potentially valuable insights for policymakers. Identifying the most influential authors can facilitate their recognition as experts and their involvement in the development of knowledge networks at national or global levels. Mapping research topics into categories such as basic, driving, niche, and emerging/declining can guide funding decisions toward either well-established areas with demonstrated potential or promising emerging fields. Additionally, key research topics can be disseminated to the general public through awareness-raising campaigns, enhancing cybersecurity literacy and preparedness.

## 6 Contributions, implications and conclusions

As synthetized in Table 1, previous studies provide an overview of the use of AI, ML, and DL in phishing detection, in the form of systematic or comprehensive reviews and bibliometric analyses. The main trends identified indicate that ML dominates phishing detection methods, while DL (DNN, CNN, RNN/LSTM) is gaining ground, achieving the highest accuracy rates. NLP is becoming essential for detecting sophisticated phishing attacks, particularly spear-phishing. Distributed architecture enables Big Data analysis and real-time phishing detection. Standardized datasets (PhishTank, Alexa, UCI) are the most commonly used, supporting model comparisons. Recent bibliometric analysis (Mutluturk and Metin, 2023) reveal the constant growth of research, the strengthening of collaborations, and the central role of AI. CANTINA+ (Xiang et al., 2011) and subsequent studies on URL-based detection (Sahingoz et al., 2019) are considered foundational references in the field.

The contribution of this study in comparison with previous research is presented in Table 9. The present study offers a focused bibliometric analysis dedicated exclusively to the application of AI in phishing detection, filling a gap left by previous research that either addressed phishing broadly or within the wider context of malware. By updating the temporal landscape, it highlights an significant growth of publications, with 2024 emerging as the most productive year and 2025 maintaining the upward trend. Unlike earlier studies that only mentioned DL as a promising direction, this research documents the full technological transition from classical ML to DL and hybrid models, explaining its drivers and performance advantages. Furthermore, it extends the discussion beyond academic models by incorporating practical insights from real-world AI-powered anti-phishing systems and proposes integration pathways into enterprise security infrastructures, thus bridging theoretical advancements with applied cybersecurity practices.

**Table 9:** Contributions.

Dimension	Previous studies	Contributions of the present study	Added value compared to previous research
Scope and focus	Most studies are either general reviews (Mujtaba et al., 2017; Qabajeh et al., 2018; Kunju et al., 2019; Athulya and Praveen, 2020) or broad bibliometric analyses on malware, phishing in general, or phishing and Big Data (Mat et al., 2021; Mutluturk and Metin, 2023; Pejić-Bach et al., 2023)	Bibliometric analysis focused exclusively on AI for phishing detection, complemented by a content analysis of the 10 most cited papers in the dataset	ML and DL-centric overview of phishing detection, filling a gap in previous reviews
Temporal coverage	2021–2025	Updates the temporal landscape by showing significant growth: 2024 is the most productive year (≈2 × 2023), with 2025 continuing the upward trend	Up-to-date perspective
Technological transition to DL	DL is mentioned as promising	Documents the transition from classical ML to DL and hybrid/stacking models, explaining the drivers: scalability, zero-day detection, FP reduction, and improved accuracy	Updated technological evolution
Practical implications	Mostly focused on academic models	Integrates practical examples: Microsoft Defender, Google Workspace, Barracuda, TitanHQ, Trustifi, Abnormal Security	Presentation of the implementation layer: AI-powered, real-time, behavioral, NLP, and computer vision-based detection of phishing
Integration into enterprise security	Not identified	Proposes integration of AI-powered phishing detection into SIEM, endpoint protection, secure email gateways, and cloud-based defense systems	Bridges between theoretical research and applied cybersecurity

The findings reveal several critical research gaps that require further investigation. The most visible studies predominantly focus on traditional phishing attacks and on methods based especially on URLs analysis. The success of ML and DL in detecting phishing is still to be demonstrated in the case of less conventional phishing, targeting for example IoT sensors or voice assistants. Moreover, few studies have yet addressed explainable AI (XAI), an approach aimed at increasing transparency and trust in the functioning of the algorithms used. Understanding the reasoning behind AI-based phishing detection can enhance trust among professionals and the general public, leading to higher adoption rates. For cybersecurity professionals, XAI provides interpretable insights into model operations, allowing analysts to validate findings and adjust detection parameters as needed. This interpretability is particularly valuable in complex cases requiring nuanced judgment to differentiate between sophisticated phishing attempts and benign anomalies. By ensuring transparency in decision-making, XAI empowers analysts to make informed choices, ultimately improving the effectiveness of phishing detection systems (Nguyen et al., 2024). Regulatory frameworks increasingly require transparency and accountability in AI-driven decision-making. The European Union’s Artificial Intelligence (AI) Act establishes a harmonized legal framework for AI usage, emphasizing transparency and explainability—key aspects of XAI. Targeting high-risk systems, the legislation mandates transparency, explainability, rigorous compliance assessments, and continuous monitoring throughout the system’s lifecycle (European Union, 2024). Although anti-phishing filters are not inherently classified as high-risk systems, their integration into critical infrastructures (e.g., banking, healthcare) elevates their risk level and compliance requirements. This impacts the use of opaque models and increases solution costs, as providers must invest in certifications and evaluations to meet regulatory standards. Furthermore, scalability remains a significant concern.

Despite these issues, AI-based solutions have undeniably advanced the defense mechanisms against phishing attacks, with ML methods yielding the best results. The integration of AI-based phishing detection with user-centered strategies remains underdeveloped. The analyzed research predominantly emphasizes technical solutions, often neglecting the role of human behavior in phishing susceptibility. Effective cybersecurity strategies require a combination of automated detection and adaptive user training, yet studies addressing this intersection are scarce. The lack of user awareness and humans’ inherent curiosity in responding to tempting messages continue to represent critical challenges, fostering conditions conducive to such attacks. Consequently, organizations must prioritize comprehensive training programs that educate users on how to avoid interacting with suspicious websites and links, or, where necessary, limit their exposure to critical organizational processes. Proposed solutions should integrate automated reporting mechanisms for phishing incidents by employees, in addition to browser plugins capable of autonomously detecting potential threats before they inflict damage. AI-driven technologies with self-improving capabilities hold considerable promise in this context. Future research should prioritize the full automation of phishing attack prevention by intercepting threats before malicious links reach the end user.

In conclusion, the research field investigated, AI in phishing detection, has shown an evolutionary trend beginning in 2016. The topic is of particular interest to researchers from technical fields, such as computer science, engineering, and telecommunications. The papers were extracted from the Web of Science database and were analyzed using Bibliometrix package in Biblioshiny and VOSviewer. The results indicate that the first paper was published in 2005, and the number of publications has increased almost continuously until 2022, with only minor exceptions. In 2023, the number of publications declined, likely due to the emergence of other security threats, but a remarkable growth was observed in 2024. For 2025, the data is not conclusive, as only articles published up to August 2025 were included. The highest number of articles were published in specialized journals within the field, followed by conference proceedings. The most cited articles have been published in recent years, focusing on the use of ML algorithms to identify phishing URLs and websites based on features capable of distinguishing them from the original, authentic ones. Researchers tend to work in relatively large teams, reflecting the complexity of the subject matter. Bibliometric analysis reveals a significant trend toward ML-based phishing detection solutions. These solutions typically involve extracting discriminative features from websites and training ML models to classify them as phishing or legitimate. While ML algorithms like Support Vector Machines, Random Forest, and Decision Trees have shown promising results, researchers explore new approaches, including DL and hybrid methods.

The findings highlight the importance of feature selection and the use of diverse datasets for effective phishing detection. As phishing attacks evolve, ongoing research is essential to develop robust and adaptive detection systems.

## Data Availability

The original contributions presented in the study are included in the article/supplementary material, further inquiries can be directed to the corresponding author.
